# A Worm‐Inspired Origami Robot with Multimodal Locomotion for Adaptive Mobility in Complex Pipeline Environments

**DOI:** 10.1002/advs.75500

**Published:** 2026-04-30

**Authors:** Qiwei Zhang, Kangning Tan, Zihan He, Hongsen Pang, Yanjie Wang, Hongbin Fang, Jian Xu

**Affiliations:** ^1^ Yiwu Research Institute Fudan University Yiwu Zhejiang China; ^2^ College of Intelligent Robotics and Advanced Manufacturing State Key Laboratory of Brain Function and Disorders MOE Engineering Research Center of AI & Robotics Fudan University Shanghai China; ^3^ College of Mechanical and Electrical Engineering Jiangsu Provincial Key Laboratory of Special Robot Technology Hohai University Changzhou China

**Keywords:** multimodal locomotion, origami robot, pipe robot, soft robot, worm‐like robot

## Abstract

An origami worm‐inspired robot is developed to achieve multimodal locomotion and multifunctional operation within confined and complex pipeline environments. The robot integrates eight Yoshimura‐origami crawling modules driven by pneumatic muscles, two rolling modules with deployable flaps, and a shape‐memory alloy (SMA)‐actuated waterbomb gripper, forming a compact and modular mechatronic system. A unified gait‐generation framework enables 25 distinct locomotion gaits, including earthworm‐like peristaltic crawling (rectilinear, sidewinding, and circular), inchworm‐like two‐anchor crawling, and bidirectional wheel‐rolling. Kinematic modeling predicts performance across modes and exhibits qualitative agreement with experiments, with deviations attributed to stick–slip and frictional effects. The robot demonstrates robust mobility in a complex industrial pipeline scenario involving inclined, curved, variable‐diameter, and discontinuous pipes, as well as vertical detection and large‐diameter traversal. Coordinated actuation between the pneumatic and SMA systems allows effective grasping and swallowing‐like manipulation of objects with varied stiffness. The integrated design achieves high maneuverability, environmental adaptability, and functional versatility, providing a promising platform for inspection, detection, and maintenance tasks in constrained engineering environments.

## Graphical abstract

An origami worm‐inspired robot achieves multimodal locomotion in confined pipelines through mechatronic integration that embeds actuation, control, and communication within each origami module. Large, reversible configuration and dimensional changes enable 25 gaits synthesized by a unified framework across peristaltic, inchworm, and wheel‐rolling modes with a qualitatively predictive kinematic model. Demonstrations in industrial pipelines showcase adaptable mobility for inspection and maintenance.

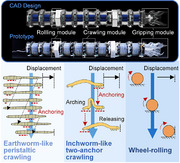

## 1 Introduction

The earthworm exhibits remarkable mobility in confined environments due to its distinctive morphology. Its body id divided by septa into numerous independently‐working segments that coordinate during locomotion. Each segment houses antagonistic muscle layers—circular and longitudinal—that drives radial contraction, axial extension, and multi‐directional bending [1]. Additionally, bristle‐like setae afford intermittent anchoring to the substrate, enabling efficient force transmission. Through retrograde peristaltic waves that orchestrate sequential anchoring and deformation, earthworms achieve versatile rectilinear, planar, and spatial locomotion. This strategy allows robot navigation across grass, sand, and subterranean soil, which provide rich bioinspiration for locomotion robots operating in narrow passages and across heterogeneous terrains.

Task demands and operating environments dictate the structural choices for earthworm‐inspired robots. Broadly, designs fall into rigid and flexible categories. Rigid segments, often realized with steel belts or acrylic plates, offer high load capacity and straightforward control at the expense of limited compliance [2, 3]. Soft segments employed continuously deformable elements such as fiber‐wound sleeves [4, 5] or silicone elastomers [6, 7], achieving superior deformability but posing greater challenges for precise control. A variety of actuators have been implemented across both classes, including shape‐memory alloy (SMA) springs [8], pneumatic actuation [9, 10, 11], servomotors [12, 13], and magnetic actuation [14]. Comparing to the rigid one, the soft segment possess excellent deformability, but is challenging to control precisely. Notably, once segmental bending is available, increasing the number and placement of actuators readily extends the repertoire from rectilinear locomotion to planar and spatial gaits [15, 16]. Equally critical is the anchoring module, the engineering analogue of setae, implemented via barbed micro‐spines, electromagnets, or negative‐pressure suction cups [17], which is indispensable for peristaltic propulsion.

Despite rapid progress, the field remains at an early stage. Many prototypes lack holistic mechatronic integration, amounting to ad hoc assemblies of structures, actuators, and controllers rather than cohesive, co‐designed systems [13, 18]. A paucity of validated application scenarios leaves many devices closer to demonstrative toys than deployable tools. Mobility, moreover, is often constrained to a narrow set of gaits, limiting adaptability to varied environments [19, 20]. For example, numerous studies have shown that earthworm‐like robots can traverse highly constrained settings—such as pipes with a diameter comparable to body size—via retrograde peristalsis and segmental anchoring [21]. However, when transitioning to open, flat terrain, the very peristaltic and anchoring mechanisms that enable pipe navigation impede speed; a five‐segment earthworm‐like robot achieves an average velocity of only 0.07 body lengths per second (BL/s) on level ground [3]. These limitations underscore the necessity of developing robots capable of switching locomotion modes on demand to match the environment. Addressing these gaps calls for the next‐generation, systematically engineered systems that incorporate truly multi‐modal locomotion capabilities for real‐world deployment.

Multi‐modal locomotion robots are single platforms endowed with two or more distinct locomotion modes, enabling adaptation to complex and changing environments [22]. Capabilities span: i) cross‐medium operation, e.g., land‐air [23, 24, 25], water‐land [26], and even water‐air transitions [27]; and ii) within medium versatility, e.g., combining crawling with jumping [28] or crawling with walking [29]. Prior work indicates two principal design routes to multi‐modality. The first is module augmentation, in which dedicated actuation/locomotion modules are added to unlock specific modes [30]; for example, outfitting a biped with a propeller to enable both walking and flight [31]. In our earlier study, we introduced active hinges on the earthworm‐like robot platform to switch between worm‐like and snake‐like gaits [32]. The second route is actuation reconfiguration, wherein a single actuation scheme supports multiple deformation patterns that, through control, yield different gaits [22, 33, 34, 35]. For instance, a pneumatically actuated segmented robot achieves inchworm‐like and gecko‐style crawling via vertical and horizontal bending, respectively [36], while a magnetoelastic micro‐robot can exhibit crawling, swimming, and jumping under a spatiotemporally programmed external magnetic field [37].

Building on these two design concepts, our goal is to expand the locomotion repertoire of earthworm‐inspired robots to strengthen environmental adaptability. In pipelines, peristaltic locomotion remains the most efficient strategy. On open, flat terrain, however, a rolling mode can substantially increase mobility efficiency; accordingly, a rolling module can be integrated into the robot design [38, 39, 40]. For interrupted or large‐diameter conduits, inchworm‐like crawling can be more effective [41]. Delivering these modes on a single robot platform demands segments with large, reversible dimensional changes, spatial deformation, and tunable stiffness. Recent advances in origami mechanics provide precisely this toolkit. Origami structures offer unconventional properties—including negative Poisson's ratio [42], programmable shape morphing [43], and multi‐stability [44, 45, 46]—that have been widely leveraged in robotics [47, 48]. For earthworm‐like robots in particular, cylindrical origami structures (e.g., the Yoshimura‐origami structure [16]) are especially suitable: it affords lightweight construction, exceptional three‐dimensional compliance, and an inherently hollow core that simplifies the integration of various actuators such as pneumatics [49] or SMA [50].

This study targets the practical demands of robotic inspection and operation within complex industrial pipelines. Typical pipeline environmental characteristics include: pipes of varying diameters, inclined sections, curved sections, vertical sections, discontinuous pipelines, and open areas outside the pipeline. Guided by these environmental constraints and based on a multi‐modal design philosophy, we engineer a mechatronically integrated platform comprising three module types: crawling, rolling, and gripping. These modules are connected in series yet are independently addressable, enabling on‐demand locomotion mode switching and gait transitions. The crawling module, which forms the primary body, integrates a Yoshimura cylindrical origami with pneumatic muscle (PM) actuators, affording large, reversible dimensional changes and spatial compliance for earthworm‐like peristalsis as well as inchworm‐like arching. The rolling module adopts a hexagonal frame with deployable flaps, also pneumatically actuated, to deliver rapid, low‐dissipation mobility on planar terrain. The gripping module leverages a waterbomb‐based origami structure, achieving stable grasping through SMA actuation.

Building upon this delicate mechatronic hardware foundation, we develop a unified gait‐generation and control framework spanning earthworm‐like, inchworm‐like, and rolling locomotion. Corresponding kinematic models are established to predict performance across modes and are validated through flat‐ground locomotion tests. We further construct a compound pipeline testbed—including level ground, inclined, curved, and vertical pipes, variable‐diameter pipes, and intentional breaks—to evaluate multi‐modal traversal, mode transition, and manipulation. These results demonstrate efficient movement across heterogeneous environments via seamless switching among gaits and modes, while grasping and environmental‐detection trials highlight the system's multi‐functionality and its promise for real‐world engineering applications.

## 2 Robot Design and Control

### 2.1 Robot Mechatronic Design and Prototype

**FIGURE 1:**
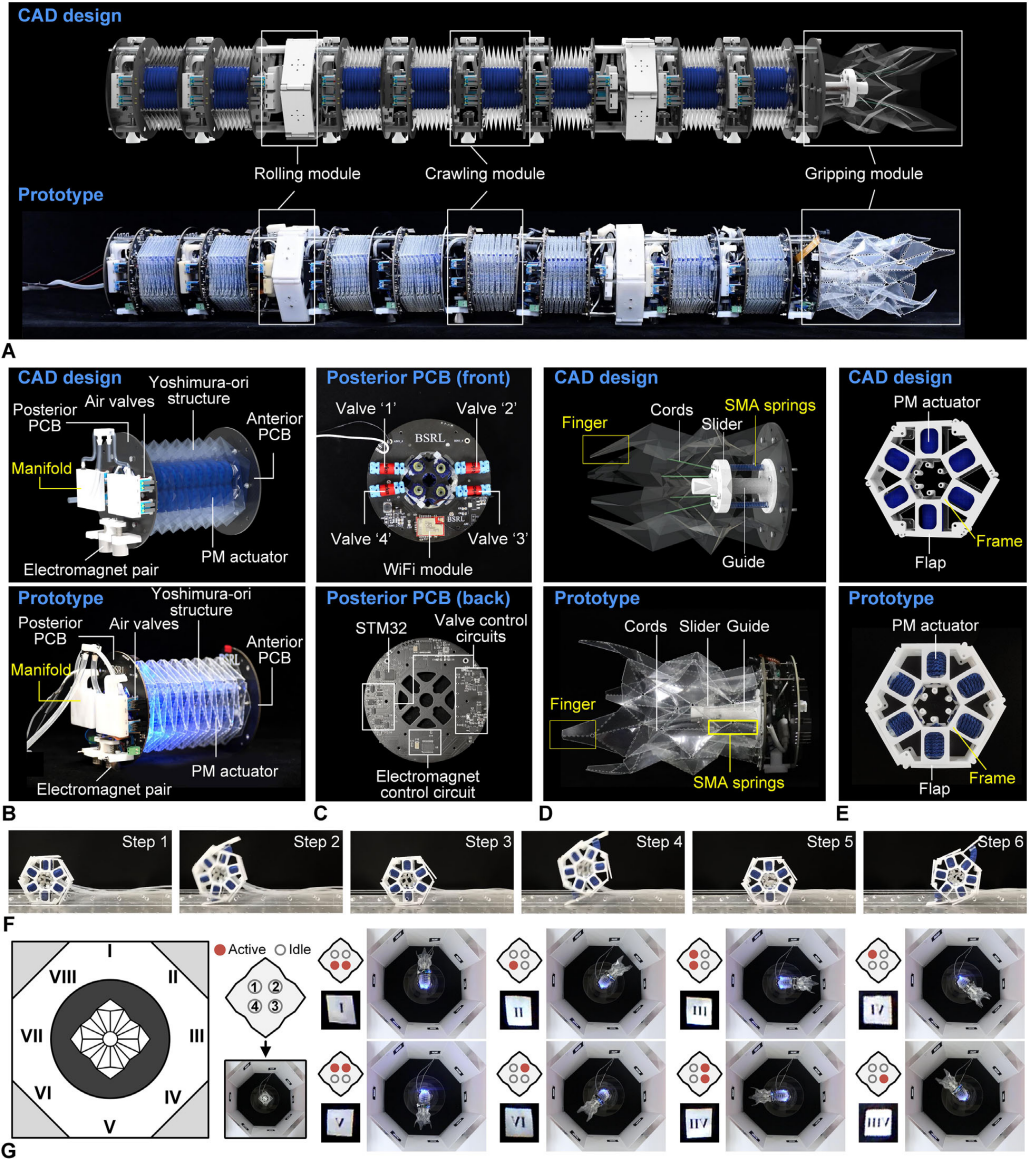
Mechatronic design and prototype of the origami worm‐like multi‐modal locomotion robot. (A) The overall CAD design and photo of the robot. (B) The CAD design and photo of the crawling module. (C) The posterior PCB, showing both the front and back sides. (D) The CAD design and photo of the gripping module. (E) The CAD design and photo of the rolling module. (F) Demonstration of the rolling module's motion. (G) Demonstration of the crawling module's deformation states.

The origami worm‐like multi‐modal locomotion robot is conceived and fabricated under a modular design principle. As shown in Figure 1A, the robot is composed of eight crawling modules, two rolling modules, and one gripping module. The crawling modules support axial extension and multi‐directional bending; the rolling modules can execute clockwise/counterclockwise rotations; and the gripping module enables object grasping.

#### 2.1.1 Crawling Module

The mechatronic layout of the crawling module is detailed in Figure 1B. Each module integrates a Yoshimura cylindrical origami core, four PM actuators, one electromagnet pair, two pneumatic manifolds, and two PCBs. The Yoshimura‐origami pattern is selected for its high axial compliance and multi‐directional reversible bending deformability (see video demonstration in Movie S1). To balance load capacity with flexibility, the Yoshimura‐origami structure is fabricated from 0.125 mm PET film (see detailed design and static characterization in Note S1 and Figure S1). Four independently valved PM actuators support axial actuation and spatial bending in multiple directions. When the four PMs are activated, the bending stiffness of the crawling module can be significantly increased from 131.61 to 913.92 N mm rad^−1^ (see detailed design and static characterization in Note S2 and Figure S2). Moreover, the Yoshimura cylindrical origami prototype exhibits excellent cyclic durability: after 600 consecutive loading cycles, the prototype maintains its mechanical integrity and shows no obvious degradation in load‐bearing performance. Specifically, the peak‐to‐peak value of the force‐time curve decreases by only 0.1% compared to the initial state (see detailed fatigue characterization in Note S2 and Figure S2). Consequently, the outstanding variable stiffness capability ensures that inactive modules maintain structural flexibility to adapt to curved pipelines, while active modules maintain sufficient stiffness to sustain specified configurations. Meanwhile, the excellent fatigue resistance of the Yoshimura‐origami structure prototype ensures stable operation of the robot under repeated actuation.

The posterior PCB hosts power electronics, a microcontroller unit (MCU) (STM32F103C8T6), a Wi‐Fi unit (EBYTE, E70‐433T14S2), and control circuits to enable local actuation control and communication with the host computer (Figure 1C). The posterior PCB is also equipped with four miniature pneumatic valves (X‐Valve, X‐2‐12‐L‐F) and an electromagnet pair, along with two pneumatic manifolds connected to the valves via short tubing to streamline the pneumatic circuit (see detailed design in Note S3 and Figure S3). The electromagnet pair primarily anchors certain modules to ferromagnetic surfaces during crawling motion. Note that the two PCBs also serve as structural interfaces to mount the origami shell and four PM actuators.

#### 2.1.2 Gripping Module

To enable robust grasping, we adopt a waterbomb‐based origami gripper mounted on a 3D‐printed slide (Figure 1D; see detailed design in Note S4 and Figure S4). A circular slider translates along the guide; four finger substructures are tethered to the slider via cords. SMA springs link the slider to the base. Upon Joule heating, SMA contraction shortens the slider‐base distance, inducing synchronized inward folding of the four fingers (see performance characterization of the SMA spring in Note S4 and Figure S4). When de‐energized, elastic recovery of the origami restores the open state. A miniature onboard camera provides local visual feedback of the surrounding environment.

#### 2.1.3 Rolling Module

The wheel is a highly efficient component invented by humans for movement, and we have integrated it seamlessly with our worm robot. As shown in Figure 1E, the rolling module consists of a 3D‐printed hexagonal frame with six rectangular flaps hinged along the frame edges. Each flap is driven at mid‐span by a compact PM actuator, and the distal edge is free. Inflation flips the flaps outward. To simplify plumbing, diagonally opposite PMs share a single valve (see detailed design in Note S5 and Figure S5). During operation (Figure 1F), the three flap pairs open in sequence, shifting the module's center of mass and producing continuous rolling. The two rolling modules are symmetrically arranged on opposite sides of the robot, with one responsible for counterclockwise rolling and the other for clockwise rolling.

#### 2.1.4 Deformation States

The deformation state of the crawling module is determined by the valve activation pattern controlling its four PM actuators. We define nine elementary states: axially‐contracted, axially‐extended, upward‐bent, rightward‐bent, leftward‐bent, downward‐bent, upper‐right‐bent, lower‐right‐bent, upper‐left‐bent, and lower‐left‐bent. The specific valve activation commands for each mode are summarized in Table S1. To demonstrate module deformability, two crawling modules and one gripping module are assembled in series and positioned within an octagonal enclosure, with its eight inner facets labeled ‘I’ through ‘VIII’. As the crawling modules execute the prescribed bending sequence at 0.2 MPa, the head‐mounted camera clearly resolved each numeral from varied viewing angles (Figure 1G), demonstrating its outstanding detection potential in confined spaces. In subsequent implementations, we exploit a reduced set of six basic states (axially‐contracted, axially‐extended, and four principal bending) to minimize control complexity while preserving maneuverability. In subsequent implementations, we exploit a reduced set of six basic states (axially‐contracted ‘0’, axially‐extended ‘1’, downward‐bent ‘2’, upward‐bent ‘3’, rightward‐bent ‘4’, and leftward‐bent ‘5’) to minimize control complexity while preserving maneuverability.

The aforementioned modules are structurally and electrically integrated in series to construct a prototype of an origami worm‐inspired multi‐modal locomotion robot. This system embodies an advanced modular design philosophy, uniting compactness with sophisticated mechatronic integration. Compared with prior origami‐based or worm‐like robotic prototypes, the presented platform represents a substantial step forward, delivering enhanced functionality, adaptability, and structural elegance within a unified architecture.

### 2.2 Control Architecture and Multi‐Modal Locomotion Gaits

The robot prototype achieves a high degree of integration among mechanical structures, electronic components, actuators, and control systems. It communicates with the host computer via Wi‐Fi, requiring only a single power supply and air source to operate all modules and execute multi‐modal locomotion and multi‐functional operations.

#### 2.2.1 Control Architecture

We design a control architecture for the robot (Note S6), with its electronic and pneumatic systems arranged according to Figure S6. Each module's PCBs, air valves, and electromagnet pairs are powered from a DC supply, while a compact air pump provides the pneumatic source to the valve manifolds. The host computer wirelessly dispatches gait commands to the MCU of each module. Each MCU i) outputs voltage signals to the valves within each module for independent control of the four PM actuators, and ii) drives current to the electromagnet for anchoring and SMA springs for gripping. A miniature camera mounted at the robot's head captures environmental images and streams them to the host computer via Wi‐Fi, enabling real‐time monitoring of scene context.

#### 2.2.2 Multi‐Modal Locomotion

**FIGURE 2:**
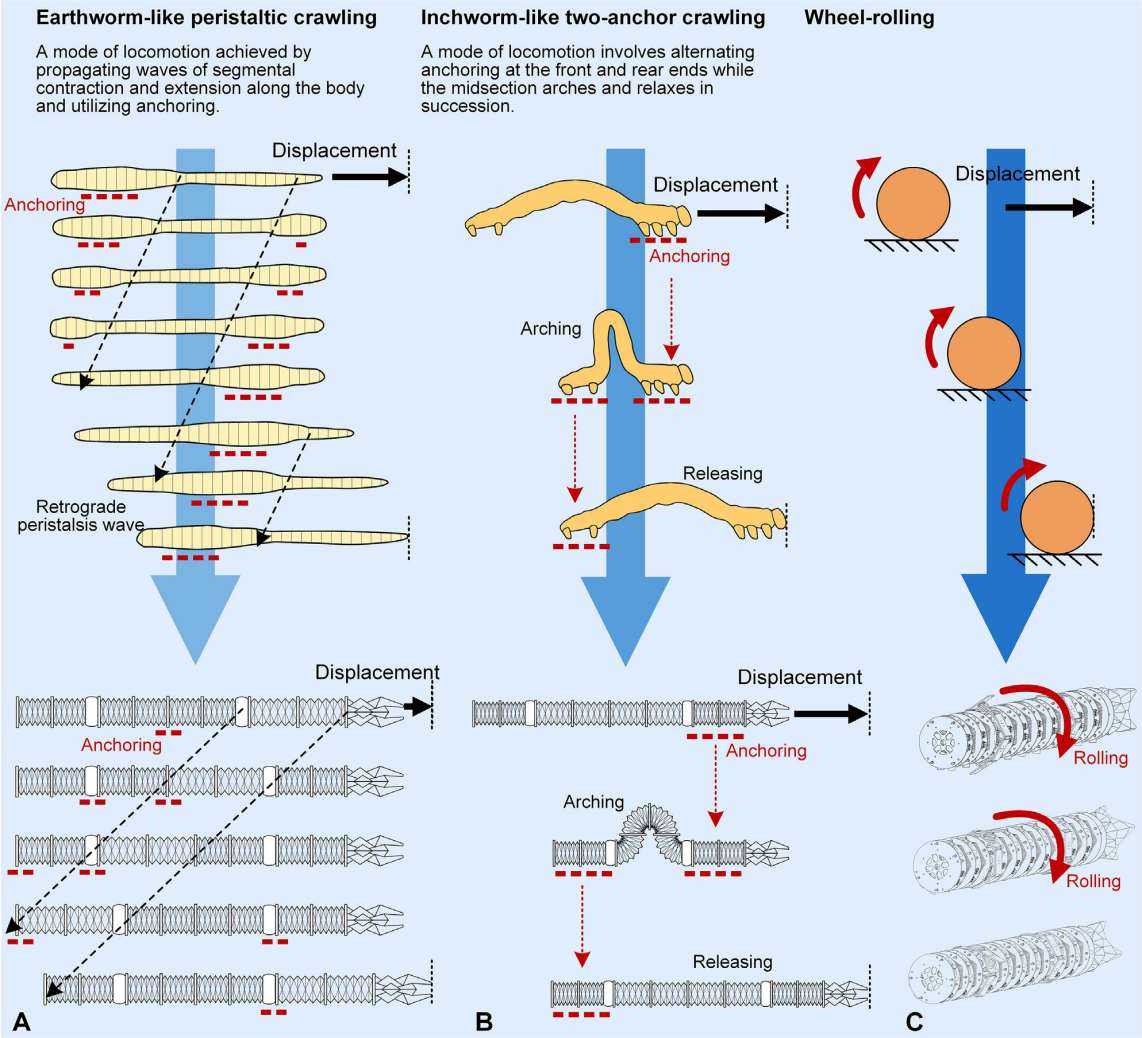
Inspirations of the robot's multi‐modal locomotion. (A) Earthworm‐like peristaltic crawling, where the anchoring (underneath dashed lines) and the retrograde peristaltic wave (dashed arrows) are illustrated. (B) Inchworm‐like two‐anchor crawling, where the loop of anchoring, arching, and releasing is demonstrated. (C) Wheel‐rolling.

The robot's excellent spatial deformability enables a repertoire of locomotion modes inspired by both biological worms and human‐engineered wheels. By combining electromagnetic anchoring with in‐plane deformation of crawling modules, the robot can reproduce earthworm‐like peristaltic crawling, achieve rectilinear, sidewinding, and circular gaits. The underlying principle follows the retrograde peristalsis wave, in which the propagation of deformation states (e.g., axial extension) occurs opposite to the direction of motion (Figure 2A). When actuated in the vertical plane, the same modules reproduce the arching gait of a caterpillar, realizing inchworm‐inspired two‐anchor crawling. In this mode, the robot first anchors its front section, releases the rear, and bends the midsection upward to form an arch, thereby drawing the tail forward. It then anchors the rear, extends the anterior body, and repeats this cyclic sequence of anchoring, arching, and releasing to achieve forward progression (Figure 2B). Finally, by sequentially actuating three pairs of flaps, the rolling module transforms the entire robot body into a wheel‐like mechanism, producing continuous lateral rolling for rapid translation across flat terrain (Figure 2C). This coordinated integration of peristaltic, inchworm, and rolling gaits demonstrates the robot's capability to perform seamless locomotion mode transitions, thereby enhancing its adaptability to diverse and unstructured environments.

#### 2.2.3 Gait Generation

Based upon the fundamental principles of the three locomotion modes described above, we establish a unified gait generation algorithm capable of synthesizing all admissible gaits. This algorithm essentially prescribes the activation sequence of the crawling module, rolling modules, and electromagnet pairs to achieve coordinated motion across different gaits. To formally define the gait, we introduce six parameters *n*_0_, *n*_1_, *n*_2_, *n*_3_, *n*_4_, and *n*_5_, representing the number of crawling modules in axially‐contracted, axially‐extended, downward‐bent, upward‐bent, rightward‐bent, and leftward‐bent states, respectively. The parameter *n_p_* denotes the number of modules whose deformation states propagating backward during a single transition step. In addition, the command variable *n*_R_ specifies the rolling direction, where values of ‘+1’ and ‘−1’ correspond to clockwise and counterclockwise rotation, respectively. Collectively, the robot's gait across all locomotion modes can be uniquely defined by parameters *G* = {*n*_0_,*n*_1_,*n*_2_,*n*_3_,*n*_4_,*n*_5_,*n*_R_|*n*_P_}. Constraints on gait parameters are provided in Note S7.

In earthworm‐like peristaltic crawling, with gait parameters *G* = {*n*_0_,*n*_1_,*n*_2_,*n*_3_,*n*_4_,*n*_5_,*n*_R_|*n*_P_} and following the law of retrograde peristalsis waves, a broad family of peristaltic gaits can be generated via the gait generation algorithm (Note S7 and Figure S7), each with distinct kinematic characteristics—including eight rectilinear gaits (‘R1–R8’ listed in Table S2; see an example in Figure 4A), six sidewinding gaits (‘S1–S6’ listed in Table S3; see an example in Figure 4C), and eight circular gaits (four right‐turning circular gaits ‘CR1–CR4’ and four left‐turning circular gaits ‘CL1–CL4’ listed in Table S4; see an example in Figure 4E). In inchworm‐like two‐anchor crawling, two effective arching gaits ‘I1’ and ‘I2’ are produced by coordinating the sequential anchoring, arching, and releasing of the front, middle, and rear sections (see an example in Figure 2B). In wheel‐rolling locomotion, only two gaits are admissible, i.e., clockwise rolling ‘RO1’ and counterclockwise rolling ‘RO2’.

### 2.3 Kinematic Modeling and Locomotion Performance Prediction

The establishment of the robot's kinematic model is based on two assumptions: 1) an energized electromagnet supplies sufficient anchoring force to prevent slip of the module; 2) the deformed module generates sufficient drive force to overcome friction and attain its commanded deformation states.

**FIGURE 3:**
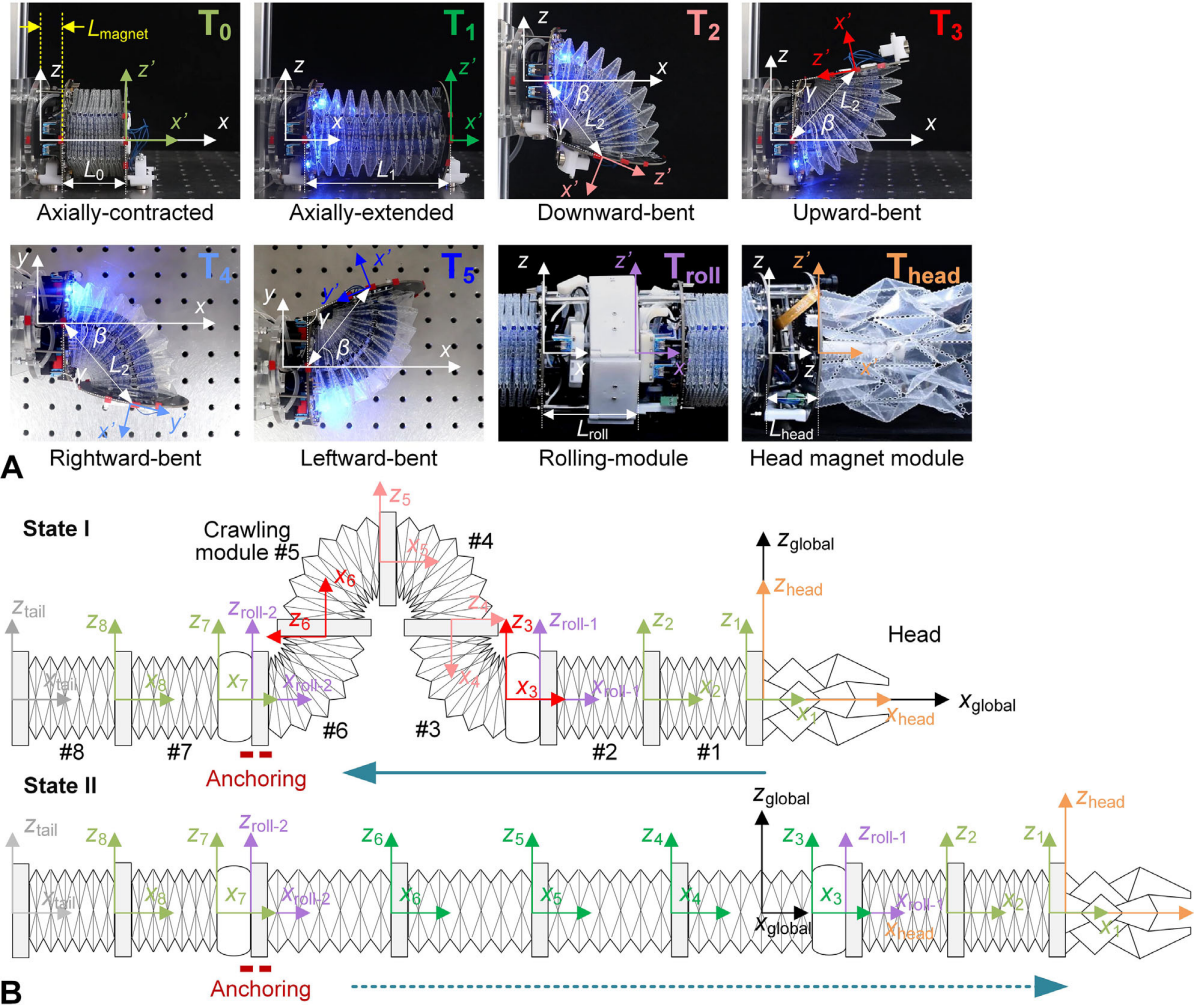
The kinematic model of the robot. (A) Frame transformations of the crawling module in different states, the rolling module, and the additional head plate. (B) Kinematic model of the robot, exemplified by a state transition (state I→state II) in inchworm‐like locomotion. The frames located on each crawling module, rolling module, and the head plate are indicated.

Robot motion is described using homogeneous transformation matrices. Specifically, each module is associated with a homogeneous transformation matrix **T**_*i*_ that maps the local frame *o‐xyz* at the center of the left end plate to the frame *o*’*‐x*’*y*’*z*’ at the center of the right end plate (Figure 3A). Here, the subscript *i* = 1,…, 8 denotes crawling modules #1 to #8; *i* = roll‐1, roll − 2 denotes the two rolling modules; *i* = head refers to the additional plate located at the head, equipped with an electromagnet pair. The detailed expressions of **T**_*i*_ are provided in Methods, matrix elements are functions of the module's kinematic and design parameters. For a crawling module, *L*_0_ and *L*_1_ denote the axial length in the axially‐contracted and axially‐extended states, respectively; *L*_2_ is the effective arc length of the crawling module in a bent state; the angle β is defined between the midpoints connection line of the right/left plates and the global *x*‐axis; γ is the bending angle of the module. These parameters depend on the driving pressure, and their specific values used for modeling are provided in Note S8. *L*_magnet_ = 22.1mm is the length of the electromagnet pair. In axially‐contracted and axially‐extended states, β = γ = 0. The fixed lengths of the rolling module and the head plate are *L*_roll_ = 51.5mm and *L*_head_ = 22.1mm.

Using the homogeneous transformation matrices, the robot's overall kinematic model is constructed, enabling quantitative description of the robot's motion during each state transition. The inchworm‐like locomotion with gait parameters *G*_I1_ = {0, 4, 2, 2, 0, 0, 0|0} is taken as an example (Figure 3B) to illustrate the pose update from State I to State II, proceeded as follows:

1) Identify the anchoring points (indicated by the red dashed lines) at state I and attach the local frame *o*_roll‐2_ − *x*_roll‐2_ *y*_roll‐2_ *z*_roll‐2_.

2) Following the blue arrows from the head to the anchoring point, the inverse transformation matrices of the intervening modules in state I are sequentially right‐multiplied to obtain the representation of the anchoring frame *o*_roll‐2_ − *x*_roll‐2_ *y*_roll‐2_ *z*_roll‐2_ in the global frame.

3) From the anchoring point to the head (blue dashed arrows), right‐multiply the forward transformation matrices of the intervening modules in State II, yielding the head frame  *o*_head_ − *x*_head_ *y*_head_ *z*_head_ in the global coordinate system after the transition.

The change of the head frame from state I to state II defines the *displacement vector* **D** of the robot; the *x*‐axis of  *o*_head_ − *x*_head_ *y*_head_ *z*_head_ defines the *heading direction* **H** of the robot. From **D** and **H**, mode and gait‐specific kinematic metrics are computed, including average velocity for all modes and gaits, and trajectory characteristics (e.g., inclination for sidewinding and turning radius for circular locomotion). Explicit formulas are provided in Methods.

## 3 Results

### 3.1 Multi‐Modal Locomotion Performance

The origami worm‐inspired robot is capable of executing 25 gaits across three locomotion modes (listed in Tables S2–S5): 23 earthworm‐like peristaltic crawling gaits (8 rectilinear, 6 sidewinding, and 8 circular), 2 inchworm‐like two‐anchor crawling gait, and 2 wheel‐rolling gaits. To demonstrate the robot's multi‐modal locomotion capabilities and validate the kinematic model, a series of experiments are conducted. The experiment setup and procedures are detailed in Note S9. During testing, the robot's head displacement **D**, heading direction **H**, and trajectory are recorded by a high‐definition camera, and the locomotion performance indexes (defined in Methods) are extracted via Kinovea video‐processing software. All crawling gaits are executed over four locomotion periods, while the rolling gaits are performed for one complete period. Representative experimental demonstrations of earthworm‐like crawling, inchworm‐like crawling, and wheel‐rolling are provided in Movies S2–S5, respectively.

#### 3.1.1 Earthworm‐Like Peristaltic Crawling

**FIGURE 4:**
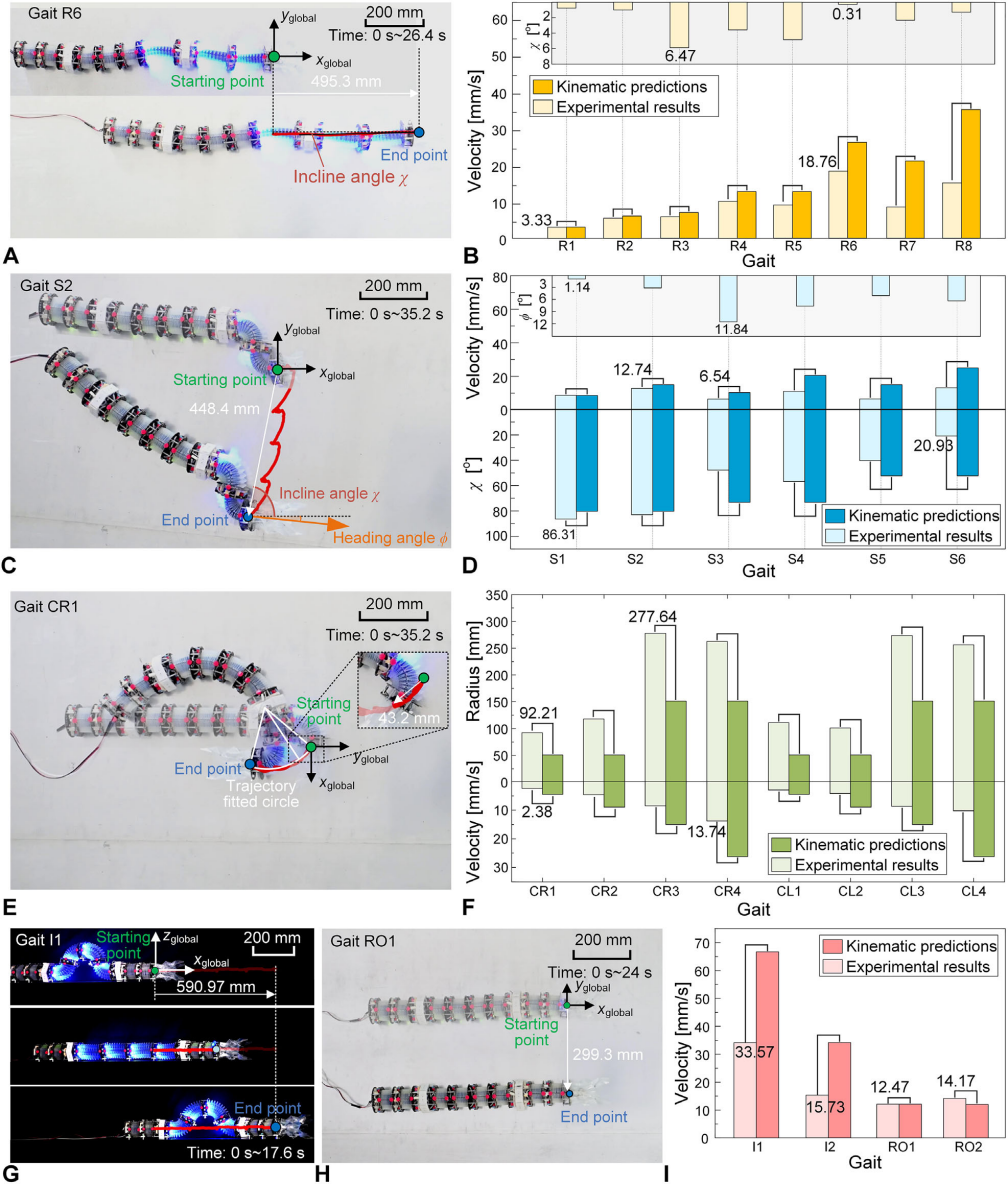
Multi‐modal locomotion tests of the robot. (A) Locomotion test under gait R6 (*G*_R6_ = {5, 3, 0, 0, 0, 0, 0|2}). (B) Locomotion performance of eight earthworm‐like rectilinear crawling gaits. (C) Locomotion test under gait S2 (*G*_S2_ = {6, 0, 0, 0, 1, 1, 0|2}). (D) Locomotion performance of six earthworm‐like sidewinding crawling gaits. (E) Locomotion test under gait CR1 (*G*_CR1_ = {7, 0, 0, 0, 1, 0, 0|1}). (F) Locomotion performance of eight earthworm‐like circular crawling giats. (G) Locomotion test under gait I1 (*G*_I1_ = {0, 4, 2, 2, 0, 0, 0|0}). (H) Locomotion test under gait RO1 (*G*_RO1_ = {8, 0, 0, 0, 0, 0, 1|0}). (I) Lowomotion perforamnce of two inchworm‐like crawling gaits and two wheel‐rolling gaits.

With different prescriptions of gait parameters, the robot demonstrates three distinct forms of peristaltic crawling locomotion—rectilinear, sidewinding, and circular—each exhibiting unique qualitative characteristics [51]. Figure 4A presents an example of rectilinear locomotion (gait R1, corresponding to gait parameters *G*_R1_ = {7, 1, 0, 0, 0, 0, 0|1}), where the starting and end points as well as the trajectory are indicated. This gait involves 8 transitions per locomotion period, with each Δ*t* lasting 2.2 s. The experimentally measured average steady‐state velocity is ${\bar{V}}_{\mathrm{Exp}-R1}=3.33\mathrm{mm}/s$, which agrees closely the kinematic prediction, exhibiting an error of only 0.59%; and the incline angle χ_Exp‐R1_ = 0.85°, indicating excellent linearity of motion. Figure 4B summarizes the experimentally obtained kinematic indices for the all rectilinear gaits and compares them with the kinematic model, with the detailed data listed in Table S2. When the number of axially‐extended modules *n*_1_ is fixed, increasing the number of propagating modules *n_p_* effectively enhances the velocity (e.g. the average stady‐state velocity of gait R4, ${\bar{V}}_{\mathrm{Exp}-R4}=10.61\mathrm{mm}/s$, exceeds that of R3, ${\bar{V}}_{\mathrm{Exp}-R3}=6.41\mathrm{mm}/s$). Furthermore, as *n*_1_ increases from 1 to 3, the average velocity rises correspondingly and reaches its maximum at *n*_1_ = 3 (gait R6), ${\bar{V}}_{\mathrm{Exp}-R3}=18.76\mathrm{mm}/s$. However, further increasing to *n*_1_ = 4 results in a velocity reduction, attributed to the excessive actuation, resulting insufficient anchoring force of the electromagnets, causing partial robot head backward slippage during locomotion (see details in Note S10). Such stick‐slip dynamic behavior, absent from the purely kinematic formulation, leads to prediction errors exceeding >30% for gaits R5–R8.

When the robot possesses equal non‐zero numbers of leftward‐bent and rightward‐bent modules (*n*_4_ = *n*_5_ ≠ 0), it performs sidewinding locomotion. For example, Figure 4C illustrates a representative example (gait S2, corresponding to gait parameters *G*_S2_ = {6, 0, 0, 0, 1, 1, 0|2}), showing the starting and end points as well as the resulting trajectory. This gait consists of four transitions per period. During a complete period, the robot produces a net linear displacement along a specific direction while maintaining an approximately constant heading direction. The experimentally measured average velocity ${\bar{V}}_{\mathrm{Exp}-S2}$ and the incline angle χ_Exp‐S2_ are 14.25 mm/s and 83.01°, respectively, with prediction errors of only 5.63% and 3.53%, confirming the validity of the kinematic model. Further analysis of all 6 sidewinding gaits reveals strong qualitative agreement between experimental and modeled results (Figure 4D). Increasing the number of propagating modules *n_p_* effectively enhances the average velocity. For example, raising *n*_P_ from 1 (gait S1) to 2 (gait S2) increases the average velocity from ${\bar{V}}_{\mathrm{Exp}-S1}=8.71\;\mathrm{mm}s^{-1}$ to ${\bar{V}}_{\mathrm{Exp}-S2}=14.25\;\mathrm{mm}s^{-1}$. On the other hand, increasing the number of axially‐extended modules *n*_1_ leads to a continual decline in the incline angle χ; when *n*_1_ increases from 0 to 1 and 2 (corresponding to gaits S2, S4, and S6), the incline angle χ decreases from 83.01° to 56.79° and 20.93°, respectively. However, similar to rectilinear locomotion, as actuation strength increases (i.e., with larger *n*_1_), slippage effects become more pronounced, leading to growing discrepancies between the experimental results and kinematic predictions, exceeding 20% and reaching 59.97% for the most strongly actuated cases, gait S6 (see details in Note S11).

When the robot possesses unequal numbers of leftward‐bent and rightward‐bent modules (*n*_4_ ≠ *n*_5_), it performs circular locomotion—executing a rightward turn when *n*_4_ > *n*_5_ or leftward turn when *n*_4_ < *n*_5_. A representative example, gait CR2 (corresponding to gait parameters *G*_CR2_ = {7, 0, 0, 0, 1, 0, 0|2}), is shown in Figure 4E, where the robot executes a rightward circular trajectory. This gait comprises four transitions per period, producing an arc‐shaped trajectory characterized by the radius of the circumscribed circle. The experimentally measured average velocity ${\bar{V}}_{\mathrm{Exp}-\mathrm{CR}2}$ and the trajectory radius *R*_Exp‐CR2_ are 4.61 mm s^−1^ and 118.04 mm, respectively. For all eight circular gaits (Figure 4F), the experimental observations show strong qualitative agreement with the kinematic predictions. Both the experimental and modeled results indicate that the maximum average velocity for rightward and leftward turning occur at gaits CR4 and CL4, respectively, and that large turning radii occur at gaits CR3, CR4, CL3, and CL4. However, certain kinematic indices exhibit notable deviations, which can be attributed to the high likelihood of slippage in both axial and lateral directions during circular motion (see details in Note S12).

It is noteworthy that during peristaltic crawling locomotion—including rectilinear, sidewinding, and circular gaits—pronounced stick–slip dynamics emerge as a key factor influencing the accuracy of kinematic predictions. In the current prototype, anchoring is achieved by electromagnets; insufficient electromagnetic force can induce undesired slippage. Although at the hardware level, using electromagnets with greater electromagnetic force could reduce slippage, this modification would increase segment mass and may compromise the upward bending deformation required for multimodal locomotion. An alternative approach is to employ a vacuum‐based anchoring method, which could further improve anti‐slippage performance [52], but at the cost of increased complexity in the pneumatic. Therefore, under the present design, both actuation dynamics and frictional interactions should be incorporated to improve modeling fidelity, underscoring the necessity of developing a comprehensive dynamic model to capture the coupled robot‐environment behavior more accurately [53].

#### 3.1.2 Inchworm‐Like Two‐Anchor Crawling

Inchworm‐like two‐anchor crawling mode provides an alternative and efficient strategy for rectilinear locomotion. Figure 4G shows the robot executing gait I1, corresponding to gait parameters *G*_I1_ = {0, 4, 2, 2, 0, 1, 0|0}. Each locomotion period consists of two transitions, with each transition interval lasting 2.2 s. Experimental measurements indicate that over four cycles, the robot achieves a total displacement of 590.97 mm, corresponding to an average velocity of 39.82 mm s^−1^, which is more than twice the maximum average velocity attained in rectilinear peristaltic crawling. This indicates that the inchworm‐like gait is particularly well suited for rapid movement across large‐diameter conduits. However, due to partial slippage of the electromagnet during anchoring, the experimental results deviate from the kinematic predictions by 49.65% and 53.84% for gaits ‘I1’ and ‘I2’, respectively (Figure 4I). This deviation further underscores the influence of adhesion performance on locomotion accuracy and highlights the importance of incorporating anchoring dynamics into future model refinements.

#### 3.1.3 Wheel‐Rolling

Wheel‐rolling represents one of the most efficient modes of human‐engineered locomotion. Incorporating this mechanism into the robot design significantly enhances its maneuverability and mobility. Figure 4H illustrates rolling locomotion corresponding to gait RO1 (with gait parameters *G*_RO1_ = {8, 0, 0, 0, 0, 0, 1|0}). The robot completes a full rotation in 24 s. Experimental measurements reveal average steady‐state velocities of 11.62 mm/s for clockwise rolling and 11.34 mm s^−1^ for counterclockwise, with small discrepancies of 3.17% and 5.50%, respectively, compared with kinematic predictions (Figure 4I). By enabling high‐speed lateral translation, wheel‐rolling serves as an effective complement to planar crawling, significantly broadening the robot's operational envelope. This hybrid integration of biologically inspired crawling and human‐inspired rolling endows the robot with superior agility and environmental adaptability across open terrain.

Overall, the detailed kinematic indices and trajectory shape for all gaits are listed in Table S6. This table also briefly summarizes the recommended application scenarios of the different locomotion modes, providing guidance for gait selection under different performance objectives and environmental constraints.

### 3.2 Validation in Complex Industrial Pipeline Scenarios

**FIGURE 5:**
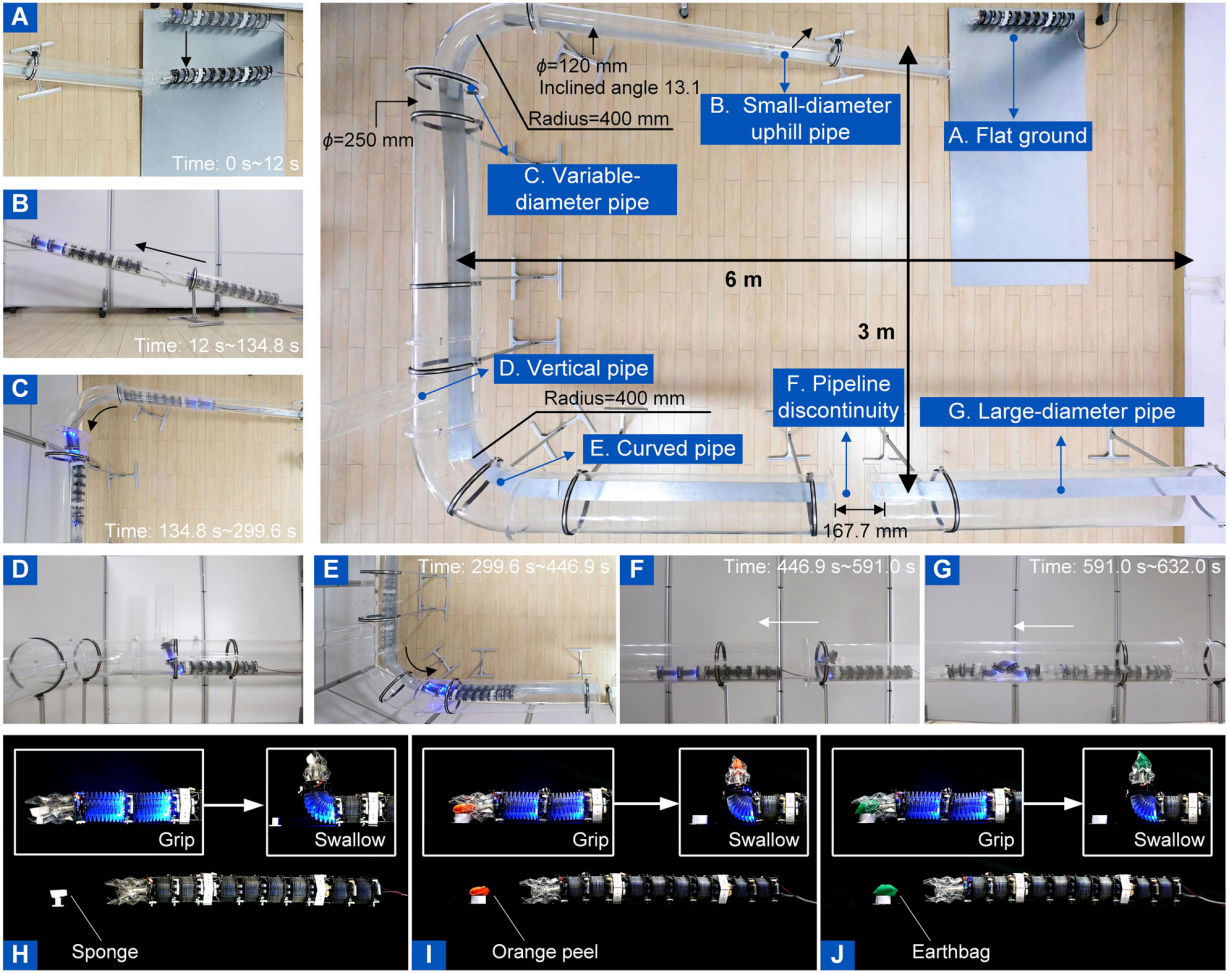
Robot field test in a complex industrial pipeline scenario. The main view displays an overview of the experimental site. A to G show snapshots of the robot's motion in different settings: (A) flat ground, (B) small‐diameter uphill pipe, (C) variable‐diameter pipe, (D) vertical pipe, (E) curved pipe, (F) pipeline discontinuity, and (G) large‐diameter pipe. (H) Gripping and swallowing of sponge, orange peel, and earthbag.

To further validate the multi‐modal locomotion capabilities of the origami worm‐like robot in realistic settings, a complex industrial pipeline scenario is constructed. The experimental site, covering an area of 3 m×6 m, incorporates multiple environments — flat ground, uphill pipe, variable‐diameter pipes, curved and vertical pipes, and discontinuities between pipes—as shown in Figure 5. The robot's movements are simultaneously captured by two high‐definition cameras, providing overhead and close‐up views. Detailed description of the setup is provided in Note S13. Based on the recorded video data, an in‐depth analysis of each locomotion scenario is conducted.

A) *Wheel‐rolling on flat ground*. On level terrain, the robot uses the wheel‐rolling mode to approach the pipe entrance, achieving a displacement of 430.7 mm within 12 s.

B) *Earthworm‐like rectilinear crawling in an inclined small‐diameter straight pipe*. In a 120 mm inner diameter pipe inclined at 13.1°, the robot employed rectilinear gait R4. The uphill slope introduces mild backward slippage, yielding a slightly reduced average velocity of 8.96 mm s^−1^, compared to its 10.61 mm s^−1^ on flat ground.

C) *Earthworm‐like crawling in a curved and variable‐diameter pipe section*. This section connects pipes with inner diameters of 120 mm and 250 mm via a curved segment (bending radius 400 mm). The compliant robot structure also accommodates curvature, allowing continuous operation using rectilinear gait R4.

D) *Erection within a vertical pipe branch*. The larger‐diameter straight pipe (inner diameter 250 mm) contains a junction to a vertical branch (inner diameter 125 mm). Crawling module #1 is actuated into an axially‐extended state, while module #2 adopts an upward‐bent state, producing a lifting motion of 194.7 mm that enables the robot head to enter the vertical pipe for inspection. The maneuver is completed in 6.8 s.

E) *Earthworm‐like crawling through a large‐diameter curved pipe*. For a curved pipe with an inner diameter of 250 mm and a bending radius of 400 mm, the robot keeps using gait R4 to traverse the curved section in 147.3 s without loss of stability, confirming its adaptability to a less confined pipe environment through structural flexibility.

F) *Traversing a pipeline discontinuity*. Pipeline discontinuity presents a fundamental challenge for conventional earthworm‐inspired robots with in‐plane deformability. Our robot, however, possesses the ability to deform and move in the vertical plane. Upon reaching a gap of 167.7 mm in the pipeline, crawling modules ‘#1’ and ‘#2’ first switch to an upward‐bent configuration, while the remaining modules maintain rectilinear locomotion (i.e. with *n*_1_ = 2, *n*_P_ = 2). After crossing the gap, modules #1 and #2 switch to axially‐extended states, guiding the head into the pipe on the opposite side. Subsequently, all modules resume rectilinear gait R4, propelling the rear modules across the discontinuity.

G) *Inchworm‐like two‐anchor crawling in a large‐diameter pipe*. Within a 250 mm‐diameter straight horizontal pipe, the robot switches to an inchworm‐like two‐anchor crawling gait I2, achieving a high velocity of 20.50 mm s^−1^, demonstrating superior performance for long‐range linear travel.

H) *Object Grasping*. Independent testing is also conducted to evaluate the robot's head‐mounted gripping module. By coordinating the pneumatic actuation of the crawling modules with the SMA‐driven deformation of the gripping module, the robot successfully achieves effective grasping and swallowing‐like motions for three distinctly different materials—sandbags, sponges, and orange peels—demonstrating its adaptability to objects of varied stiffness and surface texture.

Through all experiments, the head‐mounted camera continuously transmits real‐time visual data of the surrounding environment, facilitating precise control and monitoring. The complete experimental demonstration of the robot's motion and manipulation within the complex pipeline environment is provided in Movie S6. To supplement these field tests, additional tests are conducted to evaluate the robot's ability to navigate turns, cross gaps, and perform gripping tasks, demonstrating the limits of the robot's mobility and operational capabilities. The test results can be found in Note S14 and Movie S7.

## 4 Conclusions

We reported an origami worm‐inspired robot that integrates biologically inspired crawling mechanisms with human‐engineered rolling motion, achieving versatile locomotion within a compact, mechatronically integrated platform. Leveraging a modular architecture—eight Yoshimura‐origami crawling modules with pneumatic muscles, two pneumatically actuated rolling modules with deployable flaps, and an SMA‐driven waterbomb gripper—the system delivers large, reversible shape change, tunable stiffness, and on‐demand locomotion mode switching. A unified gait‐generation framework parameterizes and synthesizes all admissible gaits across three locomotion families, enabling 25 gaits in total: earthworm‐like rectilinear, sidewinding, and circular peristalsis; inchworm‐like two‐anchor arching; and bidirectional wheel rolling. Kinematic models can effectively predict performance under different locomotion modes and are experimentally validated. The qualitative characteristics of motion highly match experimental results, while quantitative deviations can be attributed to stick‐slip behavior and unmodeled friction/actuation dynamics.

Comprehensive trials in a complex pipeline environment—including inclined, curved, variable‐diameter, vertical, and discontinuous sections—demonstrate seamless transitions between modes and gaits, efficient traversal, and robust object grasping, while the head‐mounted camera provides continuous visual feedback. Collectively, these results establish a generalizable route to highly adaptable field robots capable of inspection and intervention in confined, heterogeneous settings.

Overall, the proposed origami worm‐inspired platform represents a significant step toward multifunctional and environment‐adaptive soft robotic systems. Its combination of modularity, mechatronic design, structural intelligence, and multimodal locomotion provides a promising foundation for future applications in pipeline inspection, search‐and‐rescue operations, and in‐situ environmental monitoring. Future work will focus on optimizing the robot's anchoring hardware and other components to further enhance its mobility and adaptability in pipeline environments. Meanwhile, it will be necessary to develop comprehensive dynamic models to systematically and quantitatively characterize the robot's performance. These efforts will contribute to advancing the robot's embodied intelligent mobility in complex real‐world environments.

## 5 Methods

### 5.1 Description of Gaits

To describe the robot state during crawling, we define the state vector of the crawling modules as $s^{t}=\left(s_{1}^{t},s_{2}^{t},s_{3}^{t},\text{…}s_{8}^{t}\right)$, where $s_{i}^{t}\in \left\{0,1,2,3,4,5\right\}$ represents the deformation state of the crawling module *i* (*i* = 1, 2, 3,…8), and the state vector of electromagnet pairs as $e^{t}=\left(e_{0}^{t},e_{1}^{t},e_{2}^{t},\text{…}e_{8}^{t}\right)$, where $e_{i}^{t}=1$ or 0 respectively denotes the ‘energized’ or ‘de‐energized’ state of the electromagnet pair *i* (*i* = 0, 1, 2, …8). Consequently, a peristaltic or two‐anchor crawling gait can be represented as a periodic sequence of **s** ^*t*^ and **e** ^*t*^. Following the underlying mechanism of each crawling locomotion mode, the key state transitions for both scenarios are derived and detailed in Note S7, with the gait generation process summarized by a flow diagram in Figure S7.

The state vector of rolling modules is defined as $R^{t}=\left(R_{1}^{t},R_{2}^{t}\right)$, where $R_{i}^{t}=1$ or 0 indicates that the rolling module is activated or inactivated. Hence, when *n*_R_ = 1, the front rolling module ‘2’ is activated ($R_{1}^{t}=0,R_{2}^{t}=1$), producing clockwise rolling (see demonstration in Figure 2C); when *n*_R_ = −1, the rear rolling module ‘1’ is activated ($R_{1}^{t}=1,R_{2}^{t}=0$), yielding counterclockwise motion. The corresponding generation process for rolling gaits is also included in the flow diagram in Figure S7.

### 5.2 Homogeneous Transformation Matrices for Robot Modules

The homogeneous transformation matrices (HTM) describe the transformations from the frame *o‐xyz* to *o*’‐*x*’*y*’*z*’. The elements in the HTM is determined from the geometries of the module. For axially‐contracted and axially‐extended modules, a translation operation is required to transform from the frame *o‐xyz* to o’‐x'y’z’, and the corresponding HTM yields

(1) $$T_{0}=\left[\begin{array}{cccc} 1 & 0 & 0 & L_{0}+L_{\mathrm{magnet}}\\ 0 & 1 & 0 & 0\\ 0 & 0 & 1 & 0\\ 0 & 0 & 0 & 1 \end{array}\right],\;T_{1}=\left[\begin{array}{cccc} 1 & 0 & 0 & L_{1}+L_{\mathrm{magnet}}\\ 0 & 1 & 0 & 0\\ 0 & 0 & 1 & 0\\ 0 & 0 & 0 & 1 \end{array}\right].$$

For downward/upward/rightward/leftward‐bent modules, the transformation involves one rotation and one translation, and the corresponding HTM (**T**_2_, downward‐bent; **T**_3_, upward‐bent; **T**_4_, rightward‐bent; **T**_5_, leftward‐bent;) can be respectively expressed as

(2) $$\begin{array}{c} T_{2}=\left[\begin{array}{cccc} \cos\gamma & 0 & -\sin\gamma & L_{2}\cos\beta +L_{\mathrm{magnet}}\\ 0 & 1 & 0 & 0\\ \sin\gamma & 0 & \cos\gamma & -L_{2}\sin\beta \\ 0 & 0 & 0 & 1 \end{array}\right],\\ T_{3}=\left[\begin{array}{cccc} \cos\gamma & 0 & \sin\gamma & L_{2}\cos\beta +L_{\mathrm{magnet}}\\ 0 & 1 & 0 & 0\\ -\sin\gamma & 0 & \cos\gamma & L_{2}\sin\beta \\ 0 & 0 & 0 & 1 \end{array}\right],\\ T_{4}=\left[\begin{array}{cccc} \cos\gamma & \sin\gamma & 0 & L_{2}\cos\beta +L_{\mathrm{magnet}}\\ -\sin\gamma & \cos\gamma & 0 & -L_{2}\sin\beta \\ 0 & 0 & 1 & 0\\ 0 & 0 & 0 & 1 \end{array}\right],\\ T_{5}=\left[\begin{array}{cccc} \cos\gamma & -\sin\gamma & 0 & L_{2}\cos\beta +L_{\mathrm{magnet}}\\ \sin\gamma & \cos\gamma & 0 & L_{2}\sin\beta \\ 0 & 0 & 1 & 0\\ 0 & 0 & 0 & 1 \end{array}\right]. \end{array}$$

For two rolling modules and the additional head electromagnet module, the transformation from frame *o‐xyz* to *o*’‐*x*’*y*’*z*’ can also be described by translation:

(3) $$T_{\mathrm{head}}=\left[\begin{array}{cccc} 1 & 0 & 0 & L_{\mathrm{head}}\\ 0 & 1 & 0 & 0\\ 0 & 0 & 1 & 0\\ 0 & 0 & 0 & 1 \end{array}\right],\;T_{\mathrm{roll}-1}=T_{\mathrm{roll}-2}=\left[\begin{array}{cccc} 1 & 0 & 0 & L_{\mathrm{roll}}\\ 0 & 1 & 0 & 0\\ 0 & 0 & 1 & 0\\ 0 & 0 & 0 & 1 \end{array}\right].$$

Based on these HTMs, the displacement vector **D** = [*D_x_*,*D_y_*,*D_z_*]^T^ and the heading direction vector **H** = [*H_x_*,*H_y_*,*H_z_*]^T^ can be derived. Without loss of generality, the robot's head frame (*o*_head_ *‐x*_head_ *y*_head_ *z*_head_) coincides with the global frame at *t*_0_ = 0s.

The displacement vector **D** and the direction vector **H** of the robot can then be obtained through multiplications of a series of HTM. Taking the inchworm‐like two‐anchor crawling (Figure 3B) as an example, **D** and **H** could be calculated by

(4) $$\begin{array}{ccc} \left[\begin{array}{c} D\\ 1 \end{array}\right] & = & T_{\mathrm{head}}^{-1}T_{0}^{-1}T_{0}^{-1}T_{\mathrm{roll}-1}^{-1}T_{3}^{-1}T_{2}^{-1}T_{2}^{-1}T_{3}^{-1}T_{1}T_{1}T_{1}T_{1}\\ & & \times \;T_{\mathrm{roll}-1}T_{0}T_{0}T_{\mathrm{head}}{\left[0,0,0,1\right]}^{T}, \end{array}$$

(5) $$\begin{array}{ccc} \left[\begin{array}{c} H\\ 0 \end{array}\right] & = & T_{\mathrm{head}}^{-1}T_{0}^{-1}T_{0}^{-1}T_{\mathrm{roll}-1}^{-1}T_{3}^{-1}T_{2}^{-1}T_{2}^{-1}T_{3}^{-1}T_{1}T_{1}T_{1}T_{1}\\ & & \times \;T_{\mathrm{roll}-1}T_{0}T_{0}T_{\mathrm{head}}{\left[1,0,0,0\right]}^{T}. \end{array}$$

### 5.3 Locomotion Performance Indexes

The robot can exhibit three qualitatively different locomotion modes: earthworm‐like peristaltic crawling, inchworm‐like two‐anchor crawling, and wheel‐rolling. Due to their distinct trajectory characteristics, different kinematic indexes are defined for locomotion performance evaluation.

The earthworm‐like peristaltic crawling further includes three types of gaits: rectilinear, sidewinding, and circular locomotion, depending on different prescriptions of gait parameters. For rectilinear locomotion, the robot does not contain any rightward, leftward, upward, or downward‐bent modules (i.e., *n*_2_ = *n*_3_ = *n*_4_ = *n*_5_ = 0). The trajectories are straight lines and can be characterized by the average velocity defined as

(6) $$\bar{V}=\frac{\left\|D\right\|}{\left(\left\lfloor \left(N_{\mathrm{total}}-n_{1}-n_{2}-n_{3}-n_{4}-n_{5}\right)/n_{p}\right\rfloor +1\right)\Delta t},$$

where ⌊ · ⌋ is the floor function, **D** is the displacement vector in a period, Δ*t* is the time required for a state transition. To further analyze the difference between the experiment and the kinematic model under rectilinear locomotion, we also defined the incline angle χ of the trajectory:

(7) $$\chi =\arccos\left(D\cdot e_{x}/\left\|D\right\|\right).$$

where **e**_*x*_ is a unit vector along the *x*‐axis of the global coordinate system. For sidewinding locomotion, the robot contains the same number of leftward‐bent and rightward‐bent modules (*n*_4_ = *n*_5_ ≠ 0). Hence, the robot can produce a net linear displacement along a specific direction while maintaining a constant heading direction. Hence, the locomotion can be characterized by the average velocity $\bar{V}$ (Equation (6)) and the incline angle χ (Equation (7)) of the trajectory. Specifically, to analyze whether the heading direction of the robot remains unchanged at the end of each period in the experiment, the heading angle ϕ is proposed herein

(8) $$\phi =\arccos\left(H\cdot e_{x}/\left\|H\right\|\right),$$

For circular locomotion, the number of leftward‐bent and rightward‐bent modules are different (*n*_4_ ≠ *n*_5_), and the robot can produce a circular trajectory. Hence, the locomotion is characterized by the average velocity $\bar{V}$ (Equation (6)) and the circumradius of the trajectory

(9) $$R=\frac{\left\|D\right\|}{2\sin\left(\arccos(H\cdot e_{x})/2\right)}.$$

For Inchworm‐like two‐anchor crawling, only linear displacement is generated, and the locomotion is evaluated by the average velocity. Note that each locomotion period consists of two transitions, and the average velocity yields

(10) $$\bar{V}=\frac{\left\|D\right\|}{2\Delta t}.$$

For wheel‐rolling locomotion, the linear displacement acquired by the robot after one complete rotation equals to the circumference of the hexagon rolling module, and the average velocity yields

(11) $$\bar{V}=\frac{l_{\mathrm{roll}}}{\Delta t},$$

where *l*_roll_ is the length of the hexagon.

## Conflicts of Interest

The authors declare no conflicts of interest.

## Supporting information




**Supporting File 1**: advs75500‐sup‐0001‐SuppMat.pdf.

**Supporting File 2**: advs75500‐sup‐0002‐MovieS1‐S7.zip.

## Data Availability

The data that support the findings of this study are available from the corresponding author upon reasonable request.
