# Supplementary material for: A Worm‐Inspired Origami Robot with Multimodal Locomotion for Adaptive Mobility in Complex Pipeline Environments
Source: Adv Sci (Weinh). 2026 Apr 30:e75500. Online ahead of print. doi: 10.1002/advs.75500 (PMC13335558; doi:10.1002/advs.75500)
Supplement: Supplementary file 1 — Supporting File 1: advs75500‐sup‐0001‐SuppMat.pdf. [file ADVS-9999-e75500-s002.pdf]

Supporting Information

A Worm-Inspired Origami Robot with Multimodal Locomotion for Adaptive Mobility in Complex Pipeline Environments

Qiwei Zhang[#], *Kangning Tan*[#], *Zihan He*, *Hongsen Pang*, *Yanjie Wang*, *Hongbin Fang*[*], and *Jian Xu*

Q. Zhang, H. Fang
Yiwu Research Institute, Fudan University, Yiwu, Zhejiang 322000, China
E-mail: fanghongbin@fudan.edu.cn (**H. Fang**)

Q. Zhang, K. Tan, Z. He, H. Pang, H. Fang, J. Xu
College of Intelligent Robotics and Advanced Manufacturing, State Key Laboratory of Brain Function and Disorders, MOE Engineering Research Center of AI & Robotics, Fudan University, Shanghai 200433, China

Y. Wang
College of Mechanical and Electrical Engineering, Jiangsu Provincial Key Laboratory of Special Robot Technology, Hohai University, Changzhou 213022, China

# These two authors contributed equally to this work.

This file includes:

- Notes S1 to S14
- Figures S1 to S13
- Tables S1 to S6
- Captions of Movies S1 to S7

Other supplementary materials for this manuscript include the following:

- Movies S1 to S7

Supporting Notes

Note S1: Detailed design and fabrication of the Yoshimura-origami structure prototype

The Yoshimura-origami structure serves as a structural scaffold that guides the axial deformation of the pneumatic muscle (PM) actuators, thereby enhancing the overall deformability and directional control of the crawling module. The planar crease pattern of the Yoshimura-origami structure is illustrated in **Figure S1A**. Its geometry is defined by four key parameters: the number of layers in the axial direction (n), the number of units in each layer (m), the unit length (l), and the angle between the diagonal and the long side (α). The setting of these parameters needs to consider the compatibility between the geometric dimensions of the Yoshimura-origami structure and the PM actuators, while ensuring that the origami structure possesses sufficient axial and bending deformation capabilities. Considering the above comprehensively, the values of the design parameters in this paper are: n=9, m=3, α=30.0°, and l=66.0 mm. The long dashed line and short dashed line denote the “mountain” and “valley” crease of the pattern, respectively. By overlapping points A, C with A*, C*, a 3D Yoshimura-origami structure could be obtained.

Figure S1B shows the Yoshimura-origami sheet fabricated from laser-cut polyethylene terephthalate (PET) film. Small holes are cut at the vertices where multiple folds intersect to reduce or eliminate the possible stress concentration. In addition, for connecting purposes, extra connection parts are added to the top, bottom, and right sides of the crease pattern. Circular holes are cut into the top and bottom connection parts for bolting to the printed circuit boards (PCBs). Finally, the right and left sections of the crease pattern are bonded together using UV-curable adhesive, thereby forming a three-dimensional Yoshimura-origami structure.

Note S2: Static and fatigue characterization of the Yoshimura-origami structure and the crawling module

Structural stiffness of the Yoshimura-origami structure plays a critical role in balancing assembly convenience and functional performance. For a given geometric design and material type, the axial stiffness of the Yoshimura-origami structure primarily depends on the thickness of the PET film. We perform quasi-static compression and extension tests on Yoshimura-origami prototypes with varying PET-film thicknesses (0.075 mm, 0.100 mm, 0.125 mm, and 0.150 mm). For each prototype, three tests are performed. The initial lengths of the prototypes with different PET thicknesses are: 122.0 mm (0.075 mm), 127.0 mm (0.100 mm), 130.0 mm (0.125 mm), and 135.0 mm (0.150 mm), respectively. The upper and lower plates of the Yoshimura-origami structure are fixed to the loading platform and the bottom platform of the universal testing machine (Instron 5965), respectively. We first compress the prototype to 30.0 mm, then stretch it to 150.0 mm, and finally compress it to its original length, with a load speed of 0.5 mm/s. **Figure S2A** displays the averaged results from three tests for each prototype, indicating that PET-film thickness significantly affects the load-bearing capacity of the prototype. For example, the restoring force at a length of 30.0 mm grows from 1.31 N to 20.74 N when the film thickness is increased from 0.075 to 0.150 mm, representing an increase of over 14.8 times. After comprehensively evaluating the Yoshimura-origami structure deformation capacity and load-bearing capability, a design with a thickness of 0.125 mm is selected as the main structure for crawling modules.

When PMs are integrated inside the Yoshimura-origami structure into a crawling module, the overall stiffness of the structure is further enhanced. As shown in **Figure S2B**, the stiffness performance of the axially-extended crawling module is tested at an air pressure of 0.20 MPa (the initial length is 102.0 mm). When the load increases from 500.0 g to 2000.0 g, the length of the crawling module decreases from 99.9 mm to 91.6 mm, representing a decay of approximately 10%. Furthermore, quasi-static testing is also performed on the inflated crawling module: it is first compressed from its original length (102.0 mm) to 97.0 mm, then stretched back to its original length at a loading rate of 0.5 mm/s. The averaged results of three trials are presented in **Figure S2C**. The inflated crawling module exhibits a restoring force of 24.47 N at a length of 97 mm, which is nearly 19 times greater than that of an unpressurized Yoshimura-origami structure of the same length (1.31 N). This demonstrates that pressurizing the internal PMs greatly enhances the axial stiffness of the crawling module.

In addition, we also examine the bending stiffness of the crawling module via the experiment setup shown in **Figure S2D** and **Figure S2E**. The posterior PCB is fixed to the supporting structure, with the coordinate system *xoy* rigidly attached to the center of the posterior PCB. One end of the pull string is fixed to the anterior PCB at point A_1, and the other

end connects to the movable platform (point A_3) of the universal testing machine, with a loading speed of 0.5 mm/s. Point A_2 represents the center of the anterior PCB. For the crawling module with and without PMs, quasi-static extension tests are performed, during which, the deformation processes are recorded using a high-definition camera, and the videos are analyzed using Kinovea to obtain the motion trajectories of target points A_1, A_2, and A_3. This analysis determines the direction vector $\overrightarrow{A_1A_3}$ of the pulling force F (recorded by the universal testing machine), the module vector $\overrightarrow{OA_2}$, and the position vector $\overrightarrow{OA_1}$. The bending torque τ_{bend} and the bending angle β can be calculated using the following equation

$$\tau_{\text{bend}} = \overrightarrow{OA_1} \times \left(\frac{F\overrightarrow{A_1A_3}}{\left\| \overrightarrow{A_1A_3} \right\|} \right) \cdot [0,0,1]^{\mathrm{T}}, \ \beta = \arccos\left(\frac{\overrightarrow{OA_2} \cdot [-1,0,0]^{\mathrm{T}}}{\left\| \overrightarrow{OA_2} \right\|} \right), \tag{S1}$$

By averaging the experimental results of the crawling module without PMs from three tests, the relationship between the bending torque τ_{bend} and the bending angel β is shown in **Figure S2D**. The curve can be fitted using a linear function, yielding a linear bending stiffness of 131.61 N·mm/rad (the coefficient of determination $r^2 = 0.997$). **Figure S2E** shows the test results for the crawling module with inflated PMs, with a fitted linear bending stiffness of 913.92 N·mm/rad ($r^2 = 0.993$), which is nearly 7 times larger than the scenario without PMs.

In addition, we have also tested the fatigue resistance of Yoshimura-origami structure. The experiment setup fatigue is shown in **Figure S2F**. The experimental specimen is a Yoshimura-ori structure with a thickness of 0.125 mm, and its upper and lower plates are fixed to the movable and bottom platforms of the universal testing machine by screws. The initial length of the Yoshimura-ori structure is 130.0 mm. In each loading cycle, the Yoshimura-ori structure is first compressed to 30 mm and then stretched to 150.0 mm, and finally compressed to its original length, with a load speed of 4 mm/s. 600 cycles are executed, and the total test duration is 36000 s (600 min, 60 s per cycle). By analyzing the force-time history data throughout the entire test and the data over the last five cycles, we find that the constitutive properties of the structure maintain good stability. Specifically, the peak-to-peak value of the force-time curve of the 600th cycle is 13.4 N, which is slightly reduced by 0.7% compared to the 13.5 N peak-to-peak value in the 1st cycle. This result fully confirms that the fabricated Yoshimura-ori structure prototype exhibits excellent fatigue resistance performance. Overall, after 600-cycle tests, the Yoshimura-ori structure made of PET films maintained its mechanical integrity and showed no obvious degradation in load-bearing performance. The nearly unchanged cyclic response also suggests that the bonded interfaces remained reliable under the present testing conditions.

Note S3: Detailed design of the pneumatic muscle and manifold

The design of the pneumatic muscle (PM) is introduced in **Figure S3A**. The PM is made up of an inner silicone tube, a 3D-printed end cap, and an outer nylon-braided sheath. One end of the silicone tube is secured to the cap, and the other end connects to an air tube; a nylon-braided sheath is wrapped around the silicone tube to restrict its radial expansion. The initial length of the PM is 45 mm.

To effectively control the pneumatic muscles, a manifold block is designed with the specific air circuit layout shown in **Figure S3B**. Two solenoid valves and two PMs (arranged vertically) are fixed on a manifold. Each solenoid valve features three ports, which connect to the positions marked by black circles in the air circuit; the PM ports are fixed at the positions indicated by orange circles. Taking a PM as an example, when the corresponding solenoid valve is energized, positive-pressure gas flows in the direction of black arrows, driving the PM into an extended state (left panel of **Figure S3B**). When the solenoid valve is de-energized, gas within the PM flows toward the environment along blue arrows, thereby inducing the PM to contract (right panel of **Figure S3B**).

The CAD design of the pneumatic system assembled on each crawling module is shown in **Figure S3C**. Two manifolds connect to the main air circuit via a four-port connector. To ensure airtightness of the manifolds, stereolithography (SLA) 3D printing technology is employed for fabrication. A U-shaped structure is designed to constrain the shape of the air tubes, preventing kinking and blockage. Furthermore, dedicated connectors are designed on the manifold to facilitate connection with PMs (**Figure S3C**). Solenoid valves are securely fixed to the manifold with screws. **Figure S3D** illustrates the connection details of the solenoid valves through the corresponding cross-sectional view. Sealing rings are installed at the interface between the solenoid valves and manifolds to prevent air leakage. A cotton layer is also needed at these interfaces as a filter to prevent solid particles from entering the solenoid valves.

Note S4: Detailed design of the waterbomb-based origami gripper

The crease pattern of the waterbomb-based origami gripper is shown in **Figure S4A**. The overall size of the structure is determined by l_w, while l_a, l_b, and l_f are used to adjust the size of fingers. To avoid interfering with the robot's movement within the pipeline, the diameter of the folded gripper should be smaller than that of the crawling module. Hence, the following geometric parameters are prescribed: $l_w = 34.0$ mm, $l_f = 27.2$ mm, $l_a = 17.0$ mm, and $l_b = 6.8$ mm. In terms of fabrication, laser cutting technology is employed to process PETE film with a thickness of 0.125 mm. Micro-perforations are also cut at the vertices where multiple creases intersect to reduce or eliminate potential stress concentration. The fabricated 2D crease-pattern sheet and the folded prototype are shown in **Figure S4B** and **S4C**, respectively. The connecting parts are used for connecting with the PCB of the gripping module.

The PCB of the gripping module is shown in **Figure S4D**. It includes two SMA power supply ports at the center, a microcontroller unit (MCU) (STM32F103C8T6), a WiFi unit (EBYTE®, E70-433T14S2), and electromagnet pair interfaces. It should be noted that the Waterbomb-based origami gripper is installed on the back of the PCB board (without any electronic components). Additionally, to achieve the robot's pipeline detection function, a 5V power supply port is provided for the video detection module (product model: ESP32-CAM).

Noting that the reliability of the gripping module is closely linked to the SMA spring, we conduct tests on the spring's recovery performance following heating. The experimental setup is shown in Figure S4E (also see below). Both ends of the SMA spring are fixed to the upper and lower plates, which are then secured to the universal testing machine with screws. The initial length of the SMA spring is 30 mm, which is the same as its original length in the gripper module. During the test, a voltage of 12 V is applied to the SMA spring for 3 s, after which the current is interrupted, and the spring is allowed to cool naturally. The test is repeated three times, and the results are averaged. The measured maximum tensile force reaches 15.2 N, providing sufficient driving force to achieve the bending motion of the finger segments in the waterbomb-based origami gripper, thereby enabling the grasping of various objects.

Note S5: Detailed design of the rolling module

The rolling module also includes a manifold, and the design of the internal air circuit is illustrated in the **Figure S5A**. In the air circuit, the orange circles represent ports for PMs, and the black circles represent ports for solenoid valves. Three solenoid valves and six pneumatic muscles are installed on this manifold. In the air circuit design of the rolling module's manifold, one solenoid valve is required to simultaneously control the states of two PMs. When the solenoid valve is energized, gas will flow towards the diagonally arranged PM ports along the black arrows to inflate and extend the PMs, causing the diagonally-arranged flaps to flip outward. When the solenoid valve is de-energized, gas in diagonally-arranged PMs will flow towards the environment along blue arrows, and the flaps will return to their original positions.

The CAD design of the rolling module's pneumatic system is shown in the **Figure S5B**. The manifold is connected to the main air circuit through a three-port connector. To ensure airtightness of the manifold, stereolithography (SLA) 3D printing technology is employed for fabrication. To facilitate connection with PMs, specialized connectors on the manifold are designed. The solenoid valves are firmly fixed to the manifold via screws. Sealing rings and filter screens are also used here.

Figure S5C shows the detailed design of the rolling module. Both the hexagonal frame and the flaps are fabricated via 3D printing. The design of PMs is the same as that of the crawling module, consisting of silicone tubes and a nylon-braided sheath.

Note S6: Control framework

The control framework for the robot is shown in **Figure S6.** Within each module, the PCB boards, solenoid valves, and electromagnet pairs are all powered by a 12 V direct current power supply. The pneumatic source for the solenoid valves is provided by an air pump. For the MCU (STM32F103C8T6) within each module, an independent control program is developed based on the module's functionality and serial number. During the control process, the host computer first wirelessly transmits gait signals to the MCU within each module. Subsequently, the MCU converts the control signal into voltages and transmits them to the solenoid valves, enabling independent control of each PM. Simultaneously, the MCU converts the control signals into current and transmits them to the electromagnet pair and the SMA springs (located in the gripping module), respectively, achieving anchoring or gripping functions. The camera is mounted on the robot head to capture images of the surrounding environment. These images are transmitted back to the host computer via WiFi, allowing operators to monitor the motion scene in real time. In terms of the specific control strategy, the operating voltage of the electromagnet is 12 V with an actuation duration of 0.2 s each time; the solenoid valve operates at 24 V and is opened for inflation for 2 s per actuation; the SMA spring in the gripping module is powered by 12 V and energized for 3 s during each grasping action.

Note S7: Multi-modal locomotion gait generation algorithm

Locomotion gaits of the robot are determined by parameters $G=\{n_0,n_1,n_2,n_3,n_4,n_5,n_R|n_P\}$. n_0, n_1, n_2, n_3, n_4, and n_5 represent the number of crawling modules in axially-contracted, axially-extended, downward-bent, upward-bent, rightward-bent, and leftward-bent states, respectively. n_P denotes the number of modules whose deformation states propagate backward during a single transition step. n_R specifies the rolling direction, which takes '+1' or '−1', corresponding to clockwise or counterclockwise rotation, respectively. Once the gait parameters are set, the algorithm can generate the corresponding locomotion gait.

To simplify the description, we first number the crawling modules and electromagnet pairs as shown in **Figure S7A**. The crawling modules are numbered sequentially from #1 to #8 from head to tail, and the electromagnet pairs are also numbered sequentially from #E0 to #E8 from head to tail. The states of crawling modules, electromagnetic pairs, and rolling modules are described by state vectors, including the state vectors for crawling modules $\mathbf{s}^t$, electromagnet pairs $\mathbf{e}^t$, and rolling modules $\mathbf{R}^t$:

$$\begin{aligned}&\mathbf{s}^t=\left(s_1^t,\ s_2^t,\ s_3^t,...\ s_8^t\right), s_i^t\in\{0,1,2,3,4,5\}, i=1,2,3...8,\\&\mathbf{e}^t=\left(e_{E0}^t,\ e_{E1}^t,\ e_{E2}^t,...\ e_{E8}^t\right), e_{Ei}^t\in\{0,1\},\ i=0,1,2...8,\\&\mathbf{R}^t=\left(R_1^t,\ R_2^t\right),\ R_i^t\in\{0,1\},\ i=1,2.\end{aligned}\tag{S2}$$

Here, s_i^t represents the deformation state of the ith crawling module at time t; e_{Ei}^t represents the 'energized' or 'de-energized' state of the ith electromagnet pair at time t ('0' represents the 'de-energized' state and '1' represents the 'energized' state); R_i^t denotes the state of the ith rolling modules at time t ('0' represents the unactivated state and '1' represents the activated state).

By inputting the gait parameters into the algorithm, the algorithm can determine the gait type and generate the corresponding locomotion gaits, which are time sequences of state vectors of crawling modules, electromagnet pairs, and rolling modules, i.e.,

$$\begin{aligned}&\left(\mathbf{s}^{t_0},\mathbf{s}^{t_0+\Delta t},......\mathbf{s}^{t_0+f\Delta t}\right),\\&\left(\mathbf{e}^{t_0},\mathbf{e}^{t_0+\Delta t},......\mathbf{e}^{t_0+f\Delta t}\right),\\&\left(\mathbf{R}^{t_0},\mathbf{R}^{t_0+\Delta t},......\mathbf{R}^{t_0+f\Delta t}\right),\end{aligned}\tag{S3}$$

where $\mathbf{s}^{t_0}$, $\mathbf{e}^{t_0}$, and $\mathbf{R}^{t_0}$ represent the initial state vectors; $\mathbf{s}^{t_0+f\Delta t}$, $\mathbf{e}^{t_0+f\Delta t}$, and $\mathbf{R}^{t_0+f\Delta t}$ represent the final state vectors. To ensure periodic locomotion, the initial state should match the final state. Subsequently, we will elaborate on the specific generation approaches for different locomotion modes and gaits (**Figure S7B**)

Earthworm-like peristaltic crawling: For earthworm-like peristaltic crawling locomotion, gait parameters n_0, n_1, n_2, n_3, n_4, n_5, and n_P need to satisfy the following constraints

$$\begin{aligned}&n_0+n_1+n_2+n_3+n_4+n_5=N_{\text{total}},\\&n_0\geq 1,n_1\geq 0,n_2\geq 0,n_3\geq 0,n_4\geq 0,n_5\geq 0,n_{\text{P}}\geq 1,n_{\text{R}}=0,\\&N_{\text{total}}>n_0+n_p,\left|n_2-n_3\right|\gamma<360^\circ,\left|n_4-n_5\right|\gamma<360^\circ,\end{aligned}\tag{S4}$$

where N_{total} is the total number of crawling modules. These constraints require: $n_{\text{P}}>1$ to achieve state propagation; the number of crawling modules should be sufficiently large to ensure at least one complete propagation of states without overlap (i.e. $N_{\text{total}}>n_0+2n_{\text{P}}$); and the robot body cannot bend beyond 360°, thereby preventing self-penetration or overlapping. Additionally, since the rolling modules do not need to be driven during crawling locomotion, the values of n_{R} are set to 0. During locomotion, the states of crawling modules are transmitted backward by n_{P} modules in each transition. To prevent rear-front switching, the number of steps f within each locomotion period is defined as follows:

$$f=\left\lfloor\left(N_{\text{total}}-n_1-n_2-n_3-n_4-n_5\right)/n_{\text{P}}\right\rfloor+1,\tag{S5}$$

where $\lfloor\cdot\rfloor$ is the floor function.

By prescribing different gait parameters that satisfy Eq. (S4), various gaits can be achieved. Specifically, for rectilinear locomotion, the following conditions need to be met:

$$n_2=n_3=n_4=n_5=0.\tag{S6}$$

For sidewinding locomotion, the following conditions need to be met:

$$n_4=n_5\neq 0\text{ and }n_2=n_3=0.\tag{S7}$$

For circular locomotion, the following conditions need to be met:

$$n_4\neq n_5\text{ and }n_2=n_3=0.\tag{S8}$$

With the abovementioned conditions, gait initialization is performed. Specifically, in the initial state, crawling modules in deformed states (i.e., states ‘1’, ‘2’, ‘3’, ‘4’, and ‘5’) are positioned at the front, and crawling modules in the same state are gathered. After gait initialization, backward propagation of crawling module states—as the fundamental mechanism underlying retrograde peristalsis—can be described by state transitions:

$$s^{t_0+j\Delta t}_{\text{Mod}[i\text{-}1+j\cdot n_{\text{P}},N_{\text{total}}]+1}=s^{t_0}_i,j=1,2,......f-1,i=1,2......N_{\text{total}},\tag{S9}$$

To achieve standardized periodic locomotion trajectories, modifications are made to the state propagation of electromagnet pairs. For both rectilinear and circular locomotion gaits, the electromagnet pair at the rear of the deformed modules is energized during the first step (i.e., $\mathbf{e}^{t_0}$); while in subsequent steps, only the electromagnet pair ‘#E0’ maintains its energized state. For sidewinding locomotion gaits, the electromagnet pair at the rear of the deformed modules also remains energized during the first step (i.e. $\mathbf{e}^{t_0}$). In subsequent steps, not only does the electromagnet pair ‘#0’ require energization, but also the energized state of the electromagnet pair at the rear of deformed modules propagates toward the tail as the states of crawling modules propagate, i.e.,

$$e^{t_0+j\Delta t}_{\text{E}(\text{Mod}[i\text{-}1+j\cdot n_{\text{P}},N_{\text{total}}]+1)}=e^{t_0}_{\text{E}i},j=1,2,......f-1,i=1,2......N_{\text{total}}.\tag{S10}$$

Inchworm-like two-anchor crawling: The fundamental locomotion mechanism of inchworm-like two-anchor crawling is as follows: the robot first anchors its front section, releases the rear, and bends the midsection upward to form an arch, thereby drawing the tail forward; it then anchors the rear, extends the anterior body, and repeats this cyclic sequence of anchoring, arching, and releasing to achieve forward progression. In our experiments, we discovered through repeated trials that only two gaits can achieve effective inchworm-like two-anchor crawling (i.e. I1 and I2). The gait parameter conditions and gait initialization are given by:

$$\begin{aligned}&\text{Gait I1}: n_2 = n_3 = 2, n_1 = 4, n_0 = 0, n_4 = n_5 = n_{\mathrm{R}} = n_{\mathrm{P}} = 0,\\&\text{Gait I2}: n_2 = 2, n_3 = 1, n_1 = 3, n_0 = 0, n_4 = n_5 = n_{\mathrm{R}} = n_{\mathrm{P}} = 0.\end{aligned} \quad \text{(S11)}$$

Since the inchworm-like two-anchor crawling gait does not require the modules to produce leftward-bent and rightward-bent deformations, and does not need to undergo module deformation state transmission, thus $n_4 = n_5 = n_{\mathrm{P}} = 0$. In addition, the rolling modules do not need to be actuated, so the value of n_{R} remains 0. The initial state settings for the two gaits are as follows

$$\text{Gait I1}: \mathbf{s}^{t_0} = (0,0,2,2,3,3,0,0), \mathbf{e}^{t_0} = (0,0,1,0,0,0,0,0), \quad \text{(S12a)}$$

$$\text{Gait I2}: \mathbf{s}^{t} = (0,0,2,3,2,0,0,0), \mathbf{e}^{t_0} = (0,0,1,0,0,0,0,0). \quad \text{(S12b)}$$

The state transitions of crawling modules and electromagnet pairs are as follows:

$$\text{Gait I1}: s_3^{t_0+\Delta t} = s_4^{t_0+\Delta t} = s_5^{t_0+\Delta t} = s_6^{t_0+\Delta t} = 1, e_{\mathrm{E2}}^{t_0+\Delta t} = 0, e_{\mathrm{E6}}^{t_0+\Delta t} = 0, \quad \text{(S13a)}$$

$$\text{Gait I2}: s_3^{t_0+\Delta t} = s_4^{t_0+\Delta t} = s_5^{t_0+\Delta t} = 1, e_{\mathrm{E2}}^{t_0+\Delta t} = 0, e_{\mathrm{E5}}^{t_0+\Delta t} = 0. \quad \text{(S13b)}$$

Wheel rolling: The fundamental locomotion mechanism of wheel rolling is as follows: the rolling module sequentially actuates three pairs of flaps, which could transform the whole robot into a wheel-like mechanism and produce continuous lateral rolling. In our experiments, two gaits are possible for wheel rolling, i.e., clockwise rolling RO1 and counterclockwise rolling RO2. The gait parameter conditions and gait initialization are given by:

$$\begin{aligned}&\text{Gait RO1}: n_0 = 8, n_{\mathrm{R}} = 1, n_1 = n_2 = n_3 = n_4 = n_5 = n_{\mathrm{P}} = 0,\\&\text{Gait RO2}: n_0 = 8, n_{\mathrm{R}} = -1, n_1 = n_2 = n_3 = n_4 = n_5 = n_{\mathrm{P}} = 0.\end{aligned} \quad \text{(S14)}$$

At the initial moment, both rolling modules are in the unactived state (i.e. $\mathbf{R}^{t_0} = (0,\ 0)$), while the subsequent state transition rules are as follows

$$\text{Gait RO1}: \mathbf{R}^{t_0+\Delta t} = (1,\ 0), \quad \text{(S15a)}$$

$$\text{Gait RO2}: \mathbf{R}^{t_0+\Delta t} = (0,\ 1). \quad \text{(S15b)}$$

The states of crawling modules and electromagnets remain ‘0’ during the wheel-rolling locomotion.

Note S8: Kinematic characterization of the crawling module

To parameterize the kinematic model, the geometric parameters of a crawling module are identified under representative deformation states. The experiment setup is shown in **Figure S8A**. Five states are evaluated—axially-extended, rightward-bent, leftward-bent, upward-bent, and downward-bent—each tested at eight driving pressures: 0.05 MPa, 0.075 MPa, 0.1 MPa, 0.125 MPa, 0.15 MPa, 0.175 MPa, 0.2 MPa, and 0.225 MPa. During each trial, inflation lasts 9.5 s, and the corresponding time histories of the deformation responses are shown in **Figure S8B**.

For the axially-extended state, the axial length of the module L_1 is used as the geometric descriptor. Pressures ≤ 0.1 MPa do not provide sufficient force to extend the crawling module. For pressure >0.1 MPa, the axial length increases monotonically with pressure, rising from 45.21 mm at 0.1MPa to 108.37 mm at 0.225 MPa.

For the bent state, we take the leftward-bent state as an example. Three kinematic parameters characterize a bent module: the effective arc length L_2, the angle between the inter-plate midpoint line and the global *x*-axis β, and the bending angle γ. As with axial-extended, pressure ≤ 0.1 MPa does not induce measurable bending. When the pressure increases from 0.1 MPa to 0.225 MPa, all three kinematic parameters increase monotonically. For example, β rises from 1.77° to 51.45°, and γ increases from 5.77° to 105.51°. At 0.2 MPa and 0.225 MPa, incremental changes become negligible (e.g., the axial length differs only slightly: 72.32 mm vs 73.38 mm; the difference in bending angle γ is also small: 99.12° vs 105.51°), indicating saturation of the deformation response. Considering performance and durability, 0.2 MPa is adopted as the driving air pressure in this study.

Since the four bending directions are theoretically symmetric, their measured parameters are averaged to obtain a single set of geometric descriptors for the deformed state. The resulting values used for modeling are: initial length $L_0 = 44.5$ mm, $L_1 = 107.1$ mm, $L_2 = 71.8$ mm, $\beta = 47.9^\circ$, and $\gamma = 99.1^\circ$.

Note S9: Experimental setup for robot locomotion tests

The experimental setup for the robot locomotion test is illustrated in **Figure S9**. During the test, 17 markers are affixed to the robot (anterior-PCB and posterior-PCB of each crawling module plus one on the gripper), labelled P_1 to P_{17} (**Figure S9A**). As shown in **Figure S9B**, the robot travels on a horizontally mounted steel plate, and the motion is recorded using two high-definition Cameras (Nikon Z7II). Camera I is arranged vertically above the testbed to capture trajectories of earthworm-like peristaltic crawling and wheel-rolling. Camera II is positioned in the plane of motion to document inchworm-like two-anchor crawling. The position data of each marker on the robot in the recorded video are analyzed using Kinovea, and the kinematic data are then calculated accordingly, including displacement, heading direction, and trajectory profiles.

i) *Displacement*. The straight-line distance between marker point P_1 (i.e., the position of the robot's head) at the initial moment and the final moment.

ii) *Average Velocity* $\overline{V}$. Displacement divided by the total time interval.

iii) *Head direction*. The direction of the vector from marker P_2 to marker P_1 (i.e., vector $\overrightarrow{P_2P_1}$) at the final moment.

iv) *Heading angle* χ. The angle between vector $\overline{P_2P_1}$ and x_{global}-axis at the final moment

v) *Incline angle* ϕ. The angle between the line segment connecting the points at the initial and final moments of the marker P_1 and the x_{global}-axis.

vi) *Radius R*. The maximum radius of the circumscribed circle of marker P_1's motion trajectory (applies only to circular locomotion).

Note S10: Experimental results for earthworm-like rectilinear locomotion

Figure S10 presents the displacement-time data for rectilinear gaits R6 ($G_{\mathrm{R6}} = \{5,3,0,0,0,0,0|2\}$) and R8 ($G_{\mathrm{R8}} = \{6,2,0,0,0,0,0|2\}$). **Figure S10A** reports the *x*-direction displacement time-histories for markers P_1, P_4, P_6, P_8, P_{10}, P_{12}, P_{14}, and P_{16} (i.e., anterior-PCB positions of each crawling module) under Gait R6. Overall, the marker trajectories follow the expected mechanism of retrograde peristaltic wave. Two error sources are, however, evident within specific state transitions:

(i) *Anchor slippage*. During the second locomotion period (6.6 s~13.2 s), the head should remain anchored in the kinematic model (6.6 s~7.6 s, dashed box), yet a backward slip of 13.1 mm is observed.

(ii) *Reduced extension under multi-module actuation*. In 11.0 s-13.2 s (shaded band), crawling modules #1~# 3 switch from the axially-contracted to the axially-extended state. The measured inter-marker distances are smaller than kinematic predictions: $\overline{P_4P_6}$ =169.8 mm (one axially-extended module + one rolling module) vs 180.7 mm predicted (10.9 mm, 6.0% error). $\overline{P_6P_8}$ =121.5 mm (one axially-extended module) vs 129.2 mm predicted (7.7 mm, 6.0% error). These deficits arise from main air-circuit flow limitations when multiple modules inflate concurrently, preventing modules from reaching their theoretical deformation.

Combined, the two factors yield a measured head displacement of 132.2 mm during this locomotion period versus 186.0 mm predicted by the kinematic model, with an absolute difference of 53.8 mm and a 28.9% relative error.

For gait R8, analysis of the second period (6.6 s~13.2 s) shows:

(i) *Anchor slippage*. The robot head exhibits a 10.0-mm backward slippage within the anchoring interval (6.6 s~7.6 s, dashed box).

(ii) *Reduced extension under multi-module actuation*. In 11.0 s~13.2 s (shaded band), crawling modules #1~#4 switch from the axially-contracted to the axially-extended state. The measured inter-marker distances are substantially smaller than kinematic predictions: $\overline{P_6P_8}$ =111.2 mm (one axially-extended module) vs 129.2 mm predicted (13.9% error).

(iii) *Anchor failure of downstream modules*. Increasing the number of simultaneously actuated modules produces a 69.4 mm backward slip at P10 during a transition when it is intended to be anchored in the kinematic model (11.0 s~13.2 s, shaded band).

These effects further reduce the performance of the robot: the measured head displacement during is 98.4 mm, which is significantly smaller than the kinematic prediction of 248.0 mm, yielding a relative error of 60.3%.

Both cases confirm that anchoring fidelity and pressure/flow-limited actuation are major reasons for discrepancies with the kinematic model. Accurate prediction of robot locomotion performance under gaits with many actuated modules will require dynamic models that incorporate air-supply dynamics, contact/friction interaction, and non-smooth stick-slip transitions at the anchors.

Note S11: Experimental results for earthworm-like sidewinding locomotion

Figure S11 shows the displacement-time histories of markers P_1, P_4, P_6, and P_8 under sidewinding gaits S4 ($G_{S4} = \{5,1,1,1,0,0,0|2\}$) and S6 ($G_{S6} = \{4,2,1,1,0,0,0|2\}$). Motion along the *x* direction exhibits clear periodicity, consistent with the propagation of the retrograde peristalsis wave.

For gait S4, the displacement-time history of P_1 (**Figure S11A** and **S11B**) indicates robust anchoring of the head with no apparent slip. In the first period (0 s~6.6 s), the head displacement (marker P_1) in *x* direction is 38.5 mm, close to the kinematic prediction 39.3 mm. By contrast, the displacement in *y* direction is 59.3 mm, far below the predicted 131.1 mm (54.7% error). The main reason is the structural compliance of the crawling module during state transitions, which prevents targets from reaching their ideal kinematic positions. As highlighted in the inset of **Figure S11A**, when crawling modules #1 and #2 (corresponding to targets $P_2 \sim P_5$) switch from leftward-/rightward-bent to axially-contracted state (shaded band), their plate midlines (the white dashed line shown in **Figure S11C**) should align with the *x*-axis; instead, both modules remain tilted toward the positive *y*-axis. For example, the *y*-coordinate of target P_4 should be 0 mm, but an offset of 28.8 mm is observed. This lateral bias reduces the subsequent negative-*y* advance and thus decreases the trajectory incline angle per period.

Increasing the number of axially-extended modules (gait S6) amplifies the above effect. In the first period (**Figure S11D)**, the head displacement in the *x* and *y* directions are 80.5 mm and 56.89 mm, respectively, versus predicted values of 101.3 mm and 131.1 mm from the kinematic model, corresponding to 20.5% and 56.6% errors. Moreover, after the robot head advances in *y* direction, a backward slip of 65.8 mm occurs during a phase when the head should remain anchored, further degrading the net lateral progression. Cumulatively, over four periods, the robot produces only 80.7 mm of lateral displacement, compared with the predicted value of 524.2 mm, exhibiting an 84.6% shortfall.

Overall, the performance of sidewinding locomotion is limited primarily by two factors: *(i) compliance-induced pose deviations* during bending-to-axial transitions and *(ii) anchoring slip* under increased actuation load. These factors reduce lateral advance and alter the effective trajectory inclination, explaining the gap to the kinematic model. Accurate prediction and improved performance require a dynamic model that incorporates module compliance, air-supply/pressure dynamics, and contact friction with stick–slip.

Note S12: Experimental results for earthworm-like circular locomotion

Figure S12 reports the head *x*-displacement and orientation (heading angle) of the robot head under circular gaits CL1 ($G_{\mathrm{CL1}} = \{7,0,1,0,0,0,0|1\}$) and CL3 ($G_{\mathrm{CL3}} = \{6,1,1,0,0,0,0|1\}$). Two mechanisms dominate trajectory errors: *(i) anchoring slippage* and *(ii) unintended heading drift* while nominally anchored.

Figure S12A plots the *x*-direction displacement-time histories of P_1, P_4, and P_6 under gait CL1. In this gait, one period contains 8 state transitions; the robot head should remain anchored during the 7 seven transitions, and only the 8-th transition would produce displacement and heading direction changes according to the kinematic model. However, in experiments, during 0~15.4 s (shaded band), the robot head (marker P_1) slips 49.4 mm in the *x*-direction. Screenshots of the robot at 0 s and 15.4 s (insets of **Figure S12A**) confirm the head no longer coincides with its initial position (the orange circle). Heading drift accompanies this slip: while nominally anchored (0~15.4 s), the head rotates −26.2° (**Figure S12B and S12C**). Prior to transiting the crawling module #1 from axially-contracted to leftward-bent, an additional −27.9° rotation occurs when the electromagnet pair is released. These rotations are absent from the kinematic model. Consequently, when the crawling module #1 completes the commanded bend, the head achieves a total instantaneous rotation of 99.9° (close to the kinematic model's 99.1°) but only a net rotation of 45.8° relative to the initial state, corresponding to an error of 53.7%. The pose error propagates to position: at the 8-th transition's end, the predicted *x*-position of the robot head is −22.6 mm, whereas the measured position in the experiment is 83.8 mm (deviation 106.32 mm). Similar slip-and-drift behavior repeats in each period, producing large departures from the predicted circular trajectory.

Figure S12D and **12E** analyze gait CL3, which comprises 7 transitions per period; the robot head should remain anchored during the first 6 transitions, and only the 7-th transition would produce displacement and heading direction changes according to the kinematic model. **Figure S12D** reveals that anchor slippage during 0~13.2 s (shaded band) is minimal. However, the head still undergoes −19.4° drift while anchored, and the heading angle reaches 29.7° after the 7-th state transition (as shown in **Figure S12E** and **12F**), versus the kinematic prediction of 99.1°, which is a 70.1% error. The heading deficit prevents the robot head from attaining the predicted position. The robot head reaches x = 56.0 mm at the 7-th transition end, while the kinematic model predicts x = −110.2 mm (deviation 166.2 mm). As a result, gait CL3 exhibits a larger trajectory error than gait CL1.

Overall, circular locomotion errors arise from electromagnet anchoring fidelity and unmodeled heading dynamics during nominally anchored phases. Accurate prediction and

control therefore require a dynamic model that incorporates anchor contact mechanics, stick–slip, and electromagnet release transients, alongside the existing kinematic framework.

Note S13: Industrial pipeline testing scenarios

A modular industrial pipeline mock-up is assembled from 5-mm-thick acrylic pipe segments (transparent for easy observation) joined by quick-connect fittings and supported by aluminum alloy frames with pipe brackets. To provide a consistent anchoring substrate for the electromagnet pairs, 2 mm-thick steel (iron) plates are installed along the inner contour of each pipe; smooth transitions are added at joints to avoid discontinuities. At the start of each trial, the robot is placed on a 3-mm-thick steel plate serving as the initial contact surface, sized to allow one full wheel-rolling period before entering the pipeline.

Scenario A*: Flat ground.* A flat terrain is set to demonstrate the robot's wheel rolling locomotion, enabling a rapid approach to the pipe entrance.

Scenario B*: Inclined small-diameter straight pipe.* An inclined straight pipe (inner diameter 120 mm, length 2000 mm, incline 13.1°) is set to test the robot's ability to climb slopes within a confined-diameter pipeline.

Scenario C*: Small-diameter 90°-curved pipe connected to a large-diameter pipe.* The uphill pipe leads into a 90°-curved pipe (bend radius 400 mm, diameter 120 mm), which further connects a larger-diameter straight section (inner diameter 250 mm). Within the confined pipe environment, the robot configuration is constrained by the pipe wall, thereby eliminating the need for active head orientation control.

Scenario D*: Large-diameter straight pipe with vertical branch.* The larger-diameter straight pipe (inner diameter 250 mm, length 1270 mm) contains a junction to a vertical branch (inner diameter 125 mm, length 500 mm). This section evaluates the robot's erection motion for vertical inspection and validates posture control during vertical entry.

Scenario E*: Large-diameter 90°-curved pipe.* After completing the vertical pipeline examination, the robot navigates through a second 90° bend with the same bending radius (400 mm). It assesses the steering performance of the robot in a less confined pipe environment.

Scenario F*: Pipeline discontinuity.* To validate the 3D traversal capability of our robot, a pipeline gap of 167.7 mm—exceeding the maximum single-module extension length—is introduced. This scenario presents a fundamental challenge for conventional earthworm-inspired robots, which are typically restricted to one or two-dimensional deformation and cannot traverse such gaps.

Scenario G*: Large-diameter horizontal pipe.* A horizontal pipe (inner diameter 250 mm, length 1500 mm) is set to evaluate the robot's high-speed inchworm-like two-anchor crawling gait, demonstrating the robot's capability to execute rapid linear motion.

Note S14: Quantitative evaluation of the robot's locomotion and task execution capabilities

To further evaluate the practical performance of the robot in representative application scenarios, additional experiments are conducted on curved-pipe traversal, crossing of pipeline discontinuities, and gripping capability.

In addition to the pipe with a bending radius of 400 mm and inner diameter of 120 mm as shown in the original manuscript (**Figure 5**), we further test the robot in a tighter curved pipe with a bending radius of 200 mm and inner diameter of 120 mm as shown in **Figure S13A**. The result shows that the robot was still able to pass through successfully. It should be noted that, in curved pipes, the robot still moves using the rectilinear locomotion gait (the gait used in this test is R1). Therefore, its ability to negotiate the bend mainly relies on the intrinsic compliance of the body rather than on a dedicated turning gait. Accordingly, the traversable pipe curvature is determined primarily by the robot's body flexibility and the pipe constraint.

Second, regarding pipeline discontinuities, we have clarified the limit under the gait used in this work as shown in **Figure S13B** and **C**. As we mentioned in the manuscript, upon reaching the gap in the pipeline, the robot first lifts the front two crawling modules #1 and #2 to approach the pipe edge. Then, the electromagnet pair #E3 would anchor, while modules #1, #2, and #3 would switch to axially-contracted, axially-extended, and axially-extended states, so that the head electromagnet pair #E0 can reach and anchor onto the opposite side of the gap, after which the robot continues forward using the rectilinear gait R4. Under this gait and the current structural configuration, the maximum crossable gap is limited by the contracted length of crawling module #1, the extended length of crawling module #2, and the extension of crawling module #3, which amounts to approximately 190 mm. As shown in **Figure S13B**, when the pipe discontinuity is 184.5 mm, the robot's head electromagnet pair #E0 successfully anchors to the opposite end of the gap during the state transitions of crawling modules #1, #2, and #3, thus enabling successful traversal. Conversely, when the discontinuity measures 205.4 mm, the robot's head electromagnet pair #E0 cannot reach the opposite end of the gap during the state transitions of crawling modules #1, #2, and #3, resulting in a failure to traverse as shown in **Figure S13C**.

Finally, we perform gripping-force tests on three representative objects with different physical properties (sponge, orange peel, and sandbag). As shown in **Figure S13D**, the object to be grasped is mounted on the bottom platform of the universal testing machine through a long screw, while the gripping module is fixed to the upper movable platform using screws. During the test, the grasped object is fixed to the long screw. For each test, the SMA springs are driven by applying 12 V for 3 s to actuate the grasping motion. After grasping, the SMA

spring is powered off, while the upper platform moves upward at 6 mm/s over a distance of 60 mm until complete separation between the gripper and the object occurred. Three repeated tests are conducted for each object, and the averaged results are presented in **Figure S13E**. The measured maximum gripping forces are approximately 0.49 N, 0.88 N, and 0.95 N, respectively. The result indicates that, under the present experimental conditions, the maximum gripping force is positively correlated with object size (i.e., 29.8 mm for the sponge, 31.2 mm for the orange peel, and 39.8 mm for the sandbag). In addition, the gravitational forces of these three objects were only 0.004 N, 0.092 N, and 0.105 N, all much smaller than the measured maximum gripping forces. These results provide direct quantitative evidence that the gripper is capable of generating stable holding forces for representative objects.

Supporting Figures

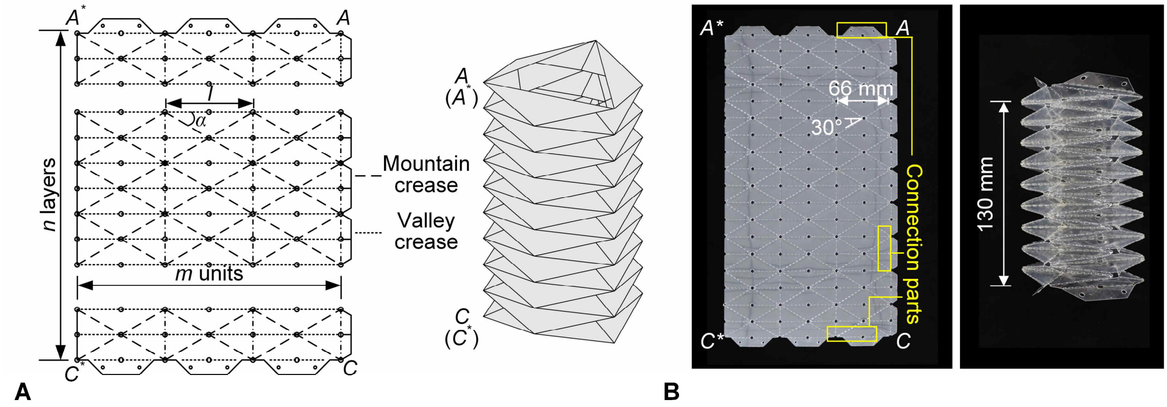

Figure S1. Detailed design and fabrication of the Yoshimura-origami structure. A) 2D crease pattern and 3D CAD model of the Yoshimura-origami structure. B) The laser-machined 3×9 Yoshimura-origami sheet made by PETE film and the obtained 3D Yoshimura-origami structure prototype.

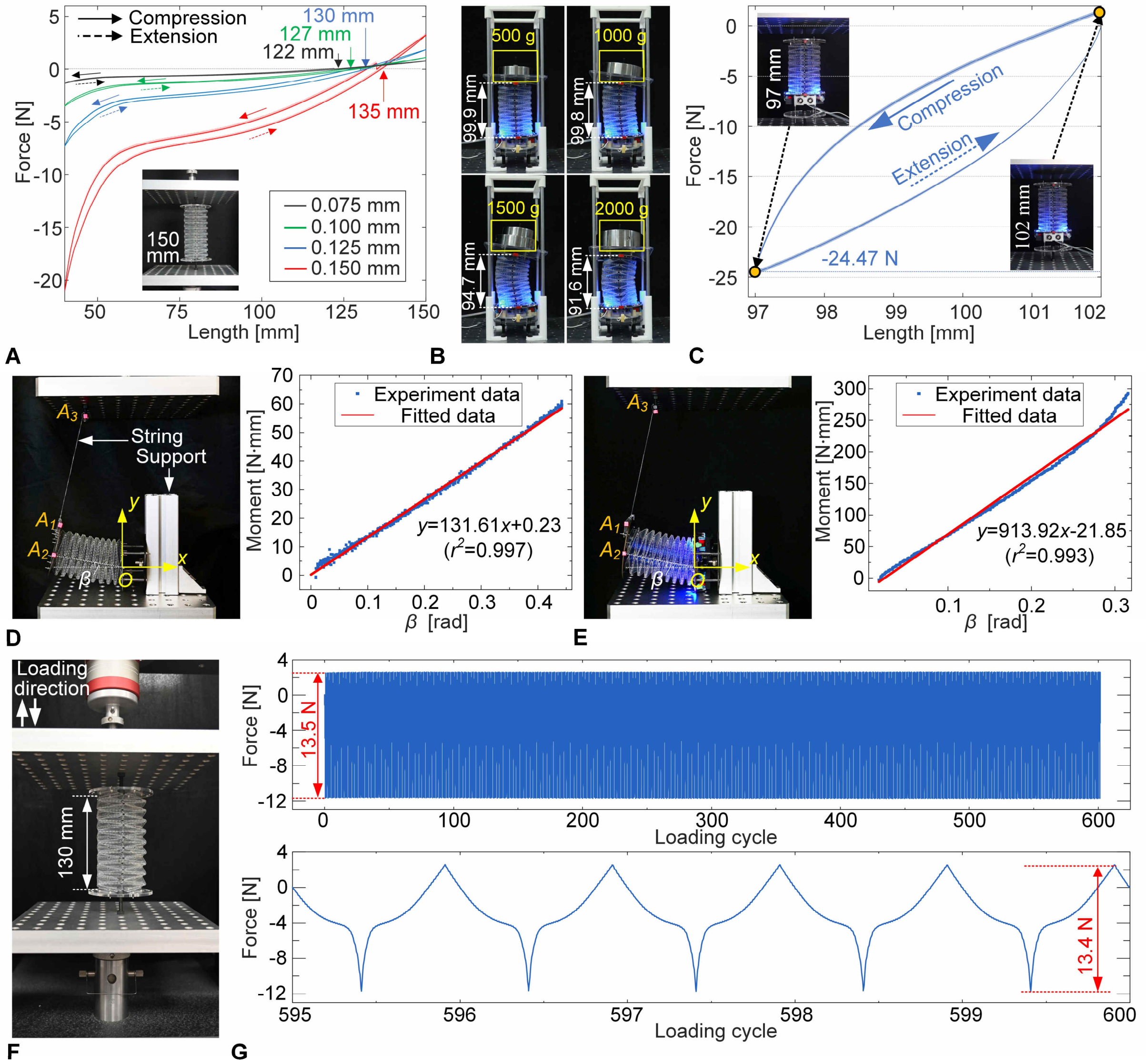

Figure S2. Static characterization of the Yoshimura-origami structure and the crawling module. A) Static characterization of Yoshimura-origami structures with different PETE film stiffness. B) Load-bearing performance test of the crawling module with inflated PMs. C) Extension and compression tests of the crawling module with inflated PMs. D) Bending test of the crawling module without PMs. E) Bending tests of the crawling module with inflated PMs. F) Experimental setup for the fatigue testing of the Yoshimura-origami structure. G) The time-force curve obtained from fatigue testing.

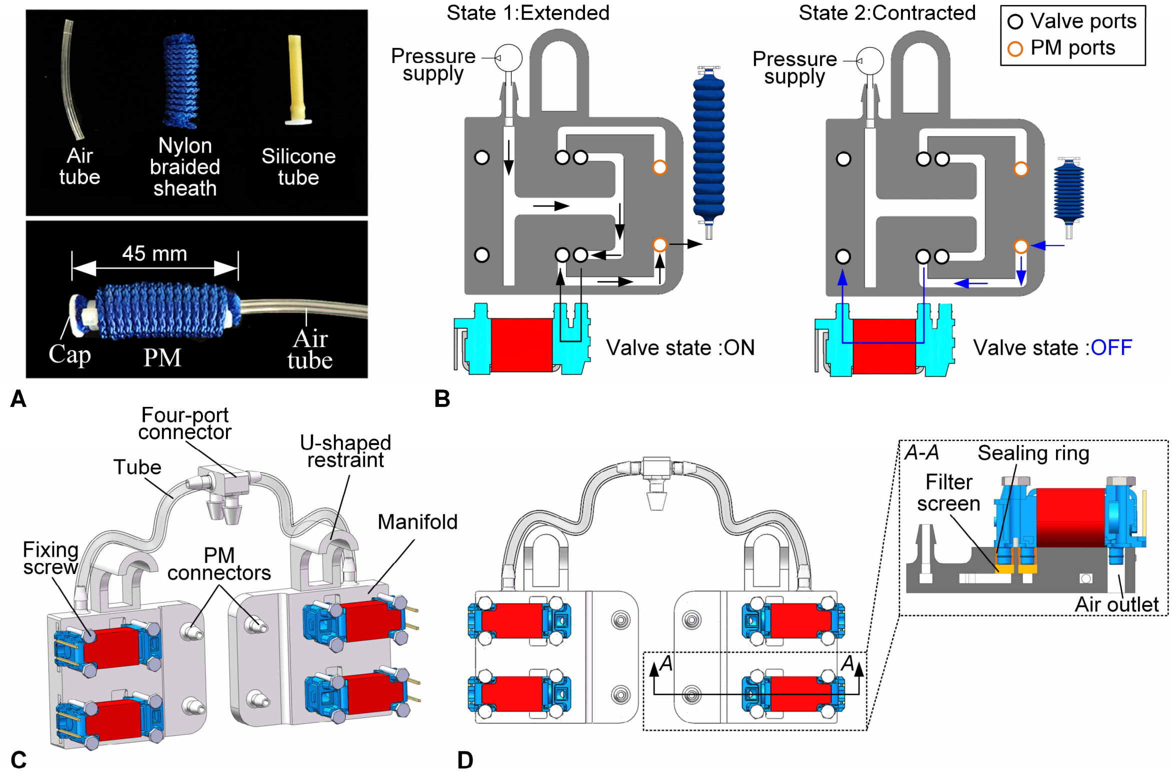

Figure S3. Design of the pneumatic muscle (PM) and manifold. A) Design and fabrication of PM. B) Using a manifold and internal air circuit to achieve elongation and contraction of PM. C) CAD design of the assembled pneumatic circuit. D) Cross-sectional view of the solenoid valve's installation position.

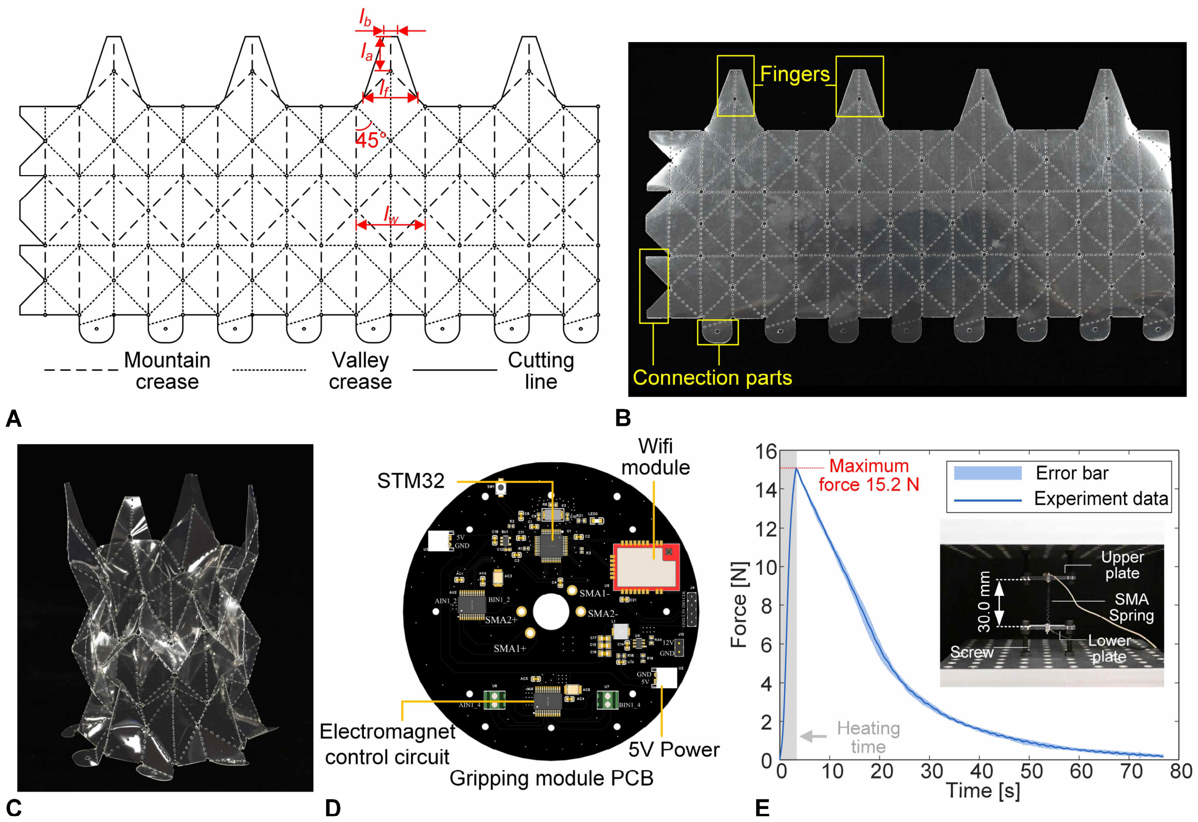

Figure S4. Design of the waterbomb-based origami gripper. A) 2D crease pattern of the waterbomb-based origami gripper. B) Laser-cut PETE sheet of the waterbomb-based origami gripper. C) Photo of the origami gripper prototype. D) Design of the gripping module's PCB. E) The experimental actuation force profile of the SMA spring.

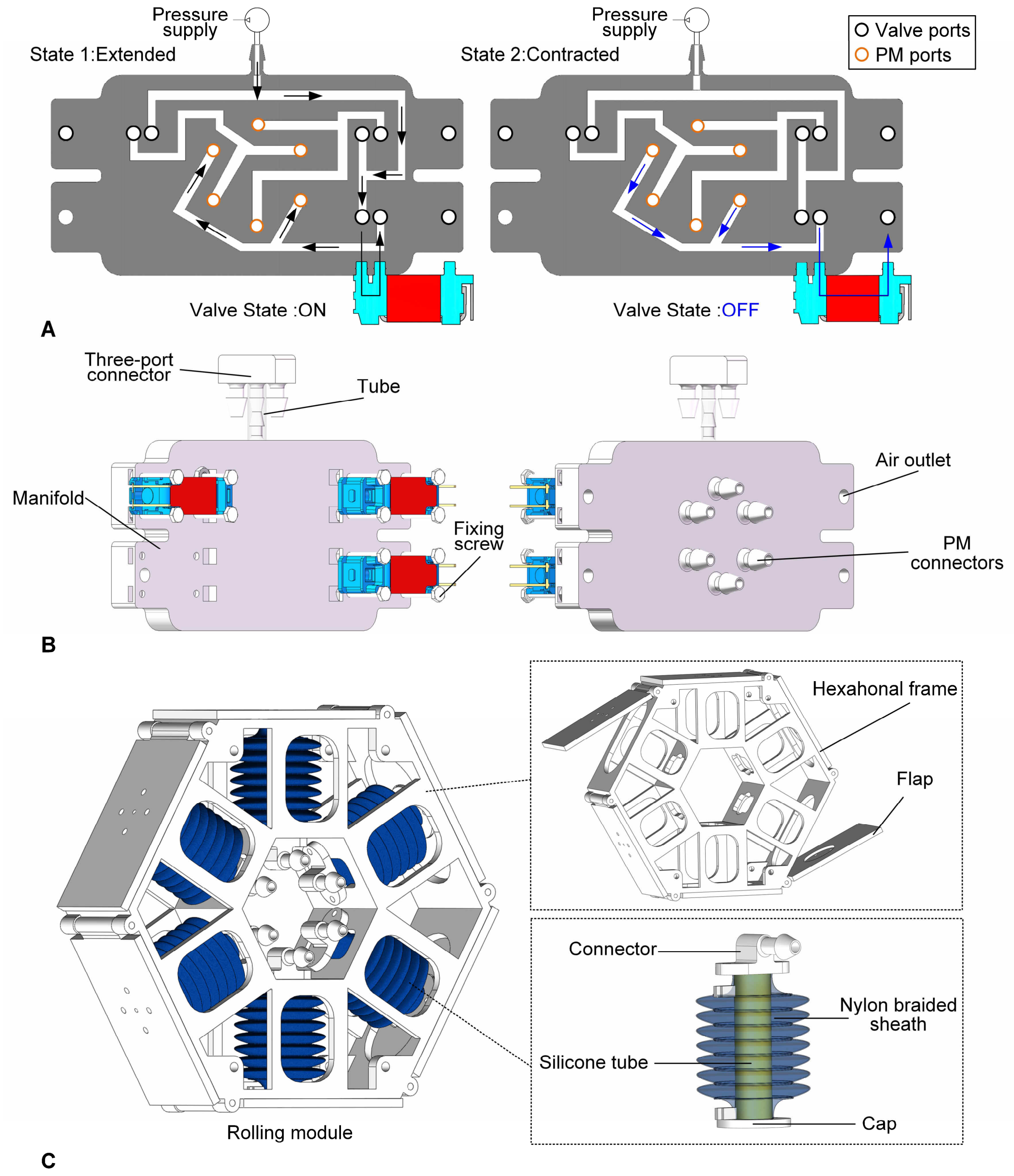

Figure S5. Detailed design of the rolling module. A) Using a manifold and internal air circuit to achieve elongation and contraction of six PMs. B) CAD design of the assembled pneumatic circuit. C) CAD design of the rolling module.

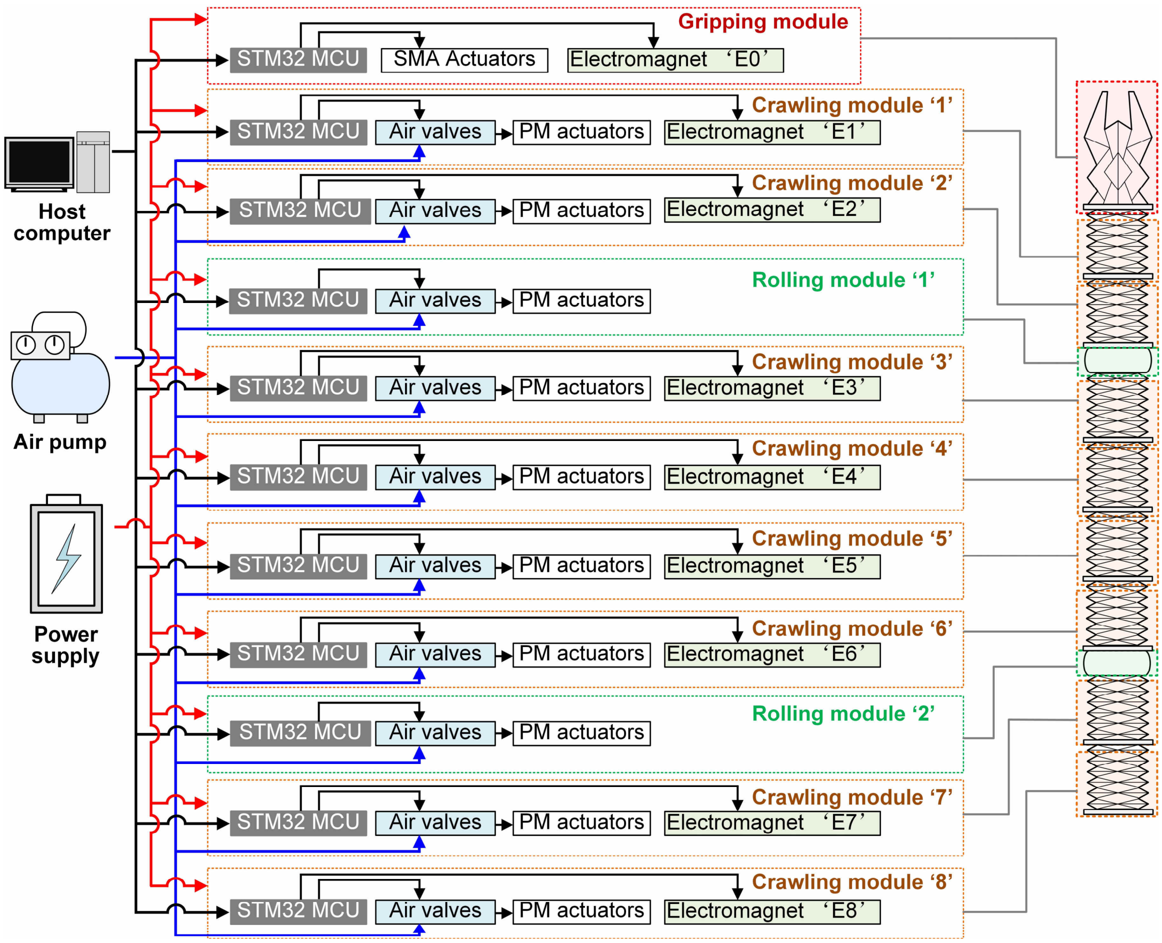

Figure S6. The control framework of the robot.

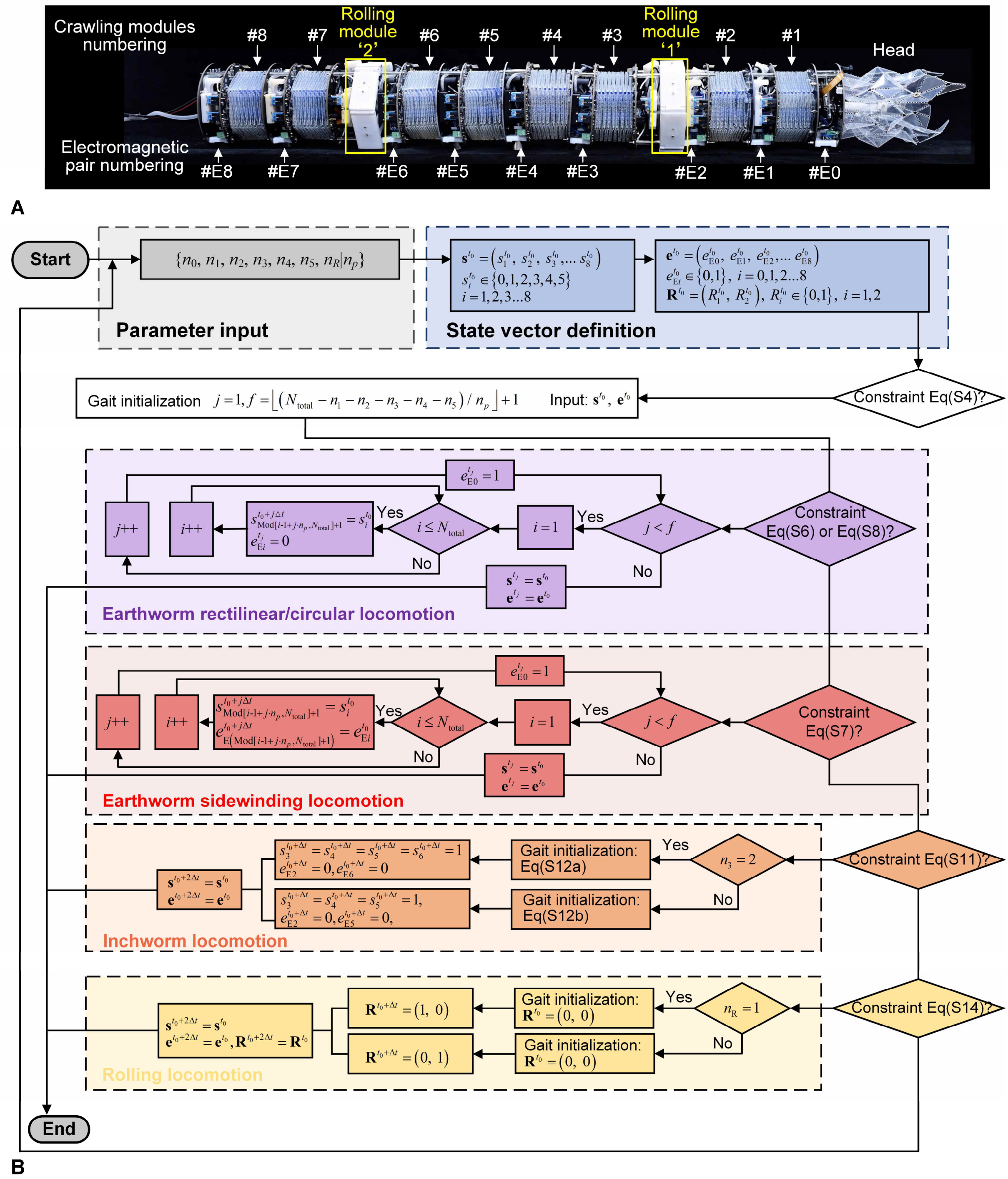

Figure S7. Generic gait generation algorithm framework for the robot. A) Numbering of crawling modules and electromagnet pairs. B) Gait generation algorithm for three locomotion modes: earthworm-like peristaltic crawling, inchworm-like two-anchor crawling, and wheel rolling.

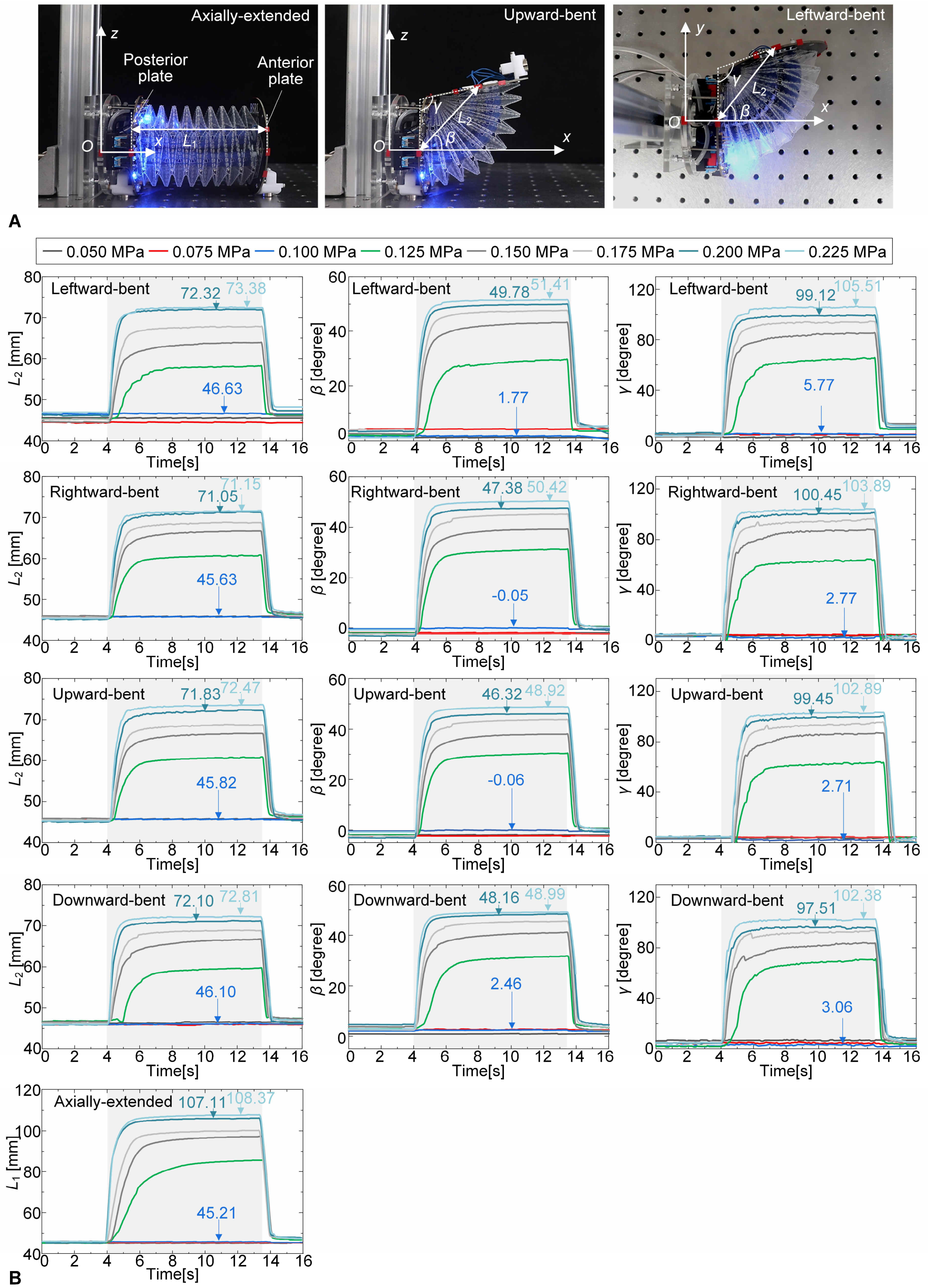

Figure S8. Deformation test of the crawling module under different driving pressures. A) The photo of the crawling module in the axially-extended, upward-bent, and leftward-bent states. B) The obtained time history of the crawling module's kinematic parameters under different deformation states.

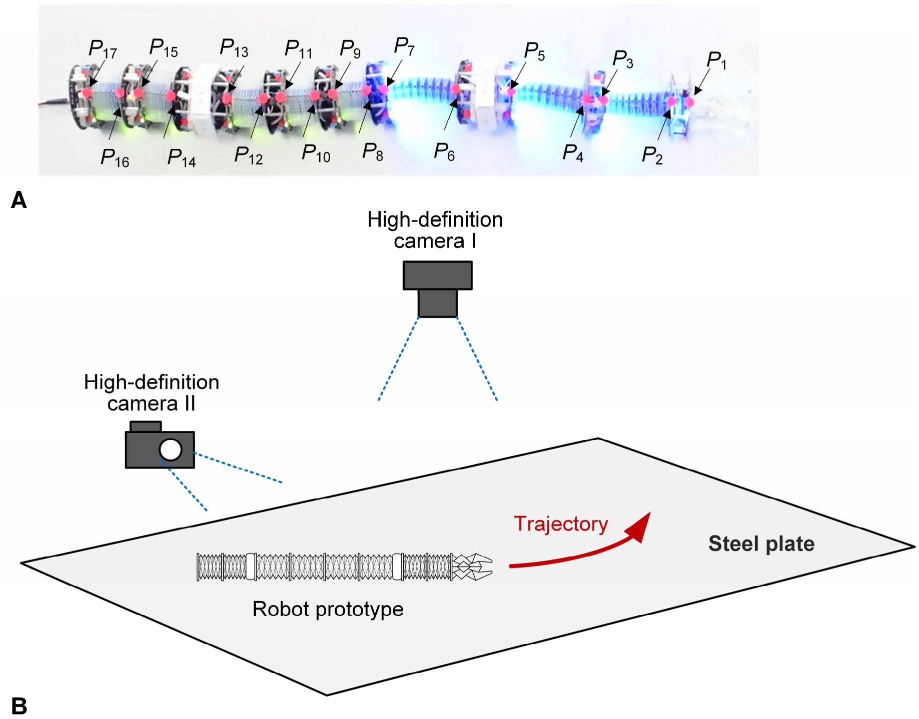

Figure S9. Experimental setup. A) Numbering of markers on the robot. B) The measurement setup for robot locomotion tests.

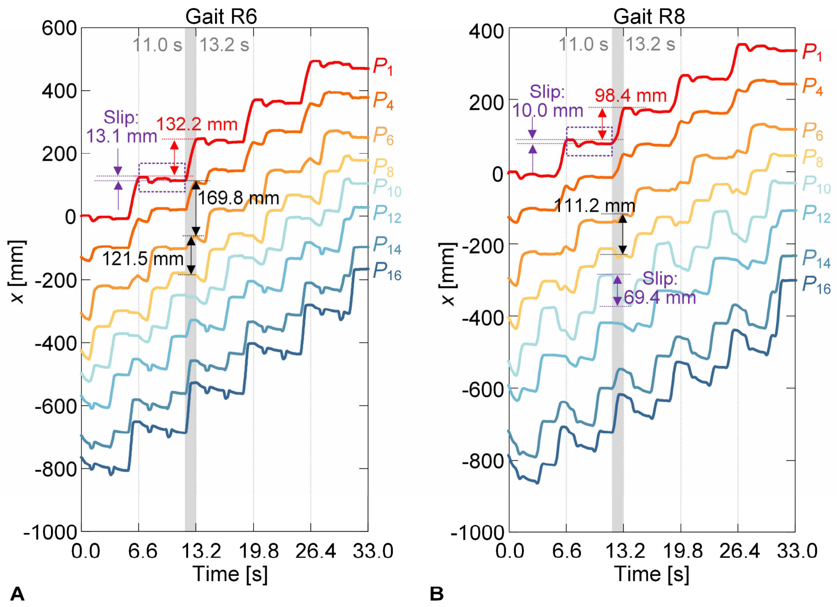

Figure S10. Experimental results of rectilinear locomotion under gaits R6 and R8. A) and B) respectively show the *x*-direction displacement-time histories of markers P_1, P_4, P_6, P_8, P_{10}, P_{12}, P_{14}, P_{16} under gaits R6 and R8.

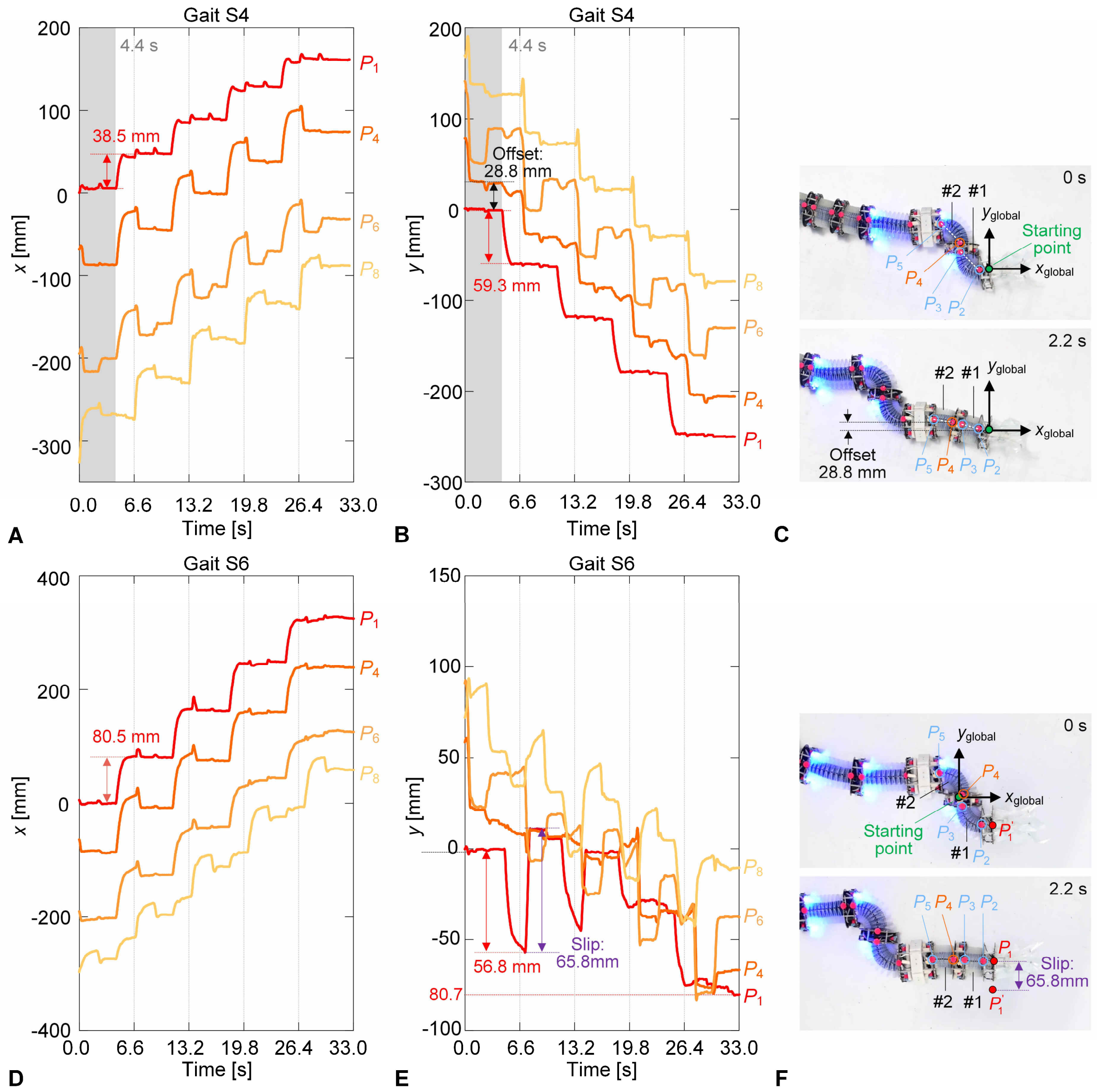

Figure S11. Experimental results of sidewinding locomotion under gaits S4 and S6. A) and B) respectively show the *x*- and *y*-direction displacement-time histories of markers P_1, P_4, P_6, and P_8 under gait S4. C) The screenshot of the robot's module displacement offset. D) and E) respectively show the *x*- and *y*-direction displacement-time histories of markers P_1, P_4, P_6, and P_8 under gait S6. F) The screenshot of the robot's head slippage.

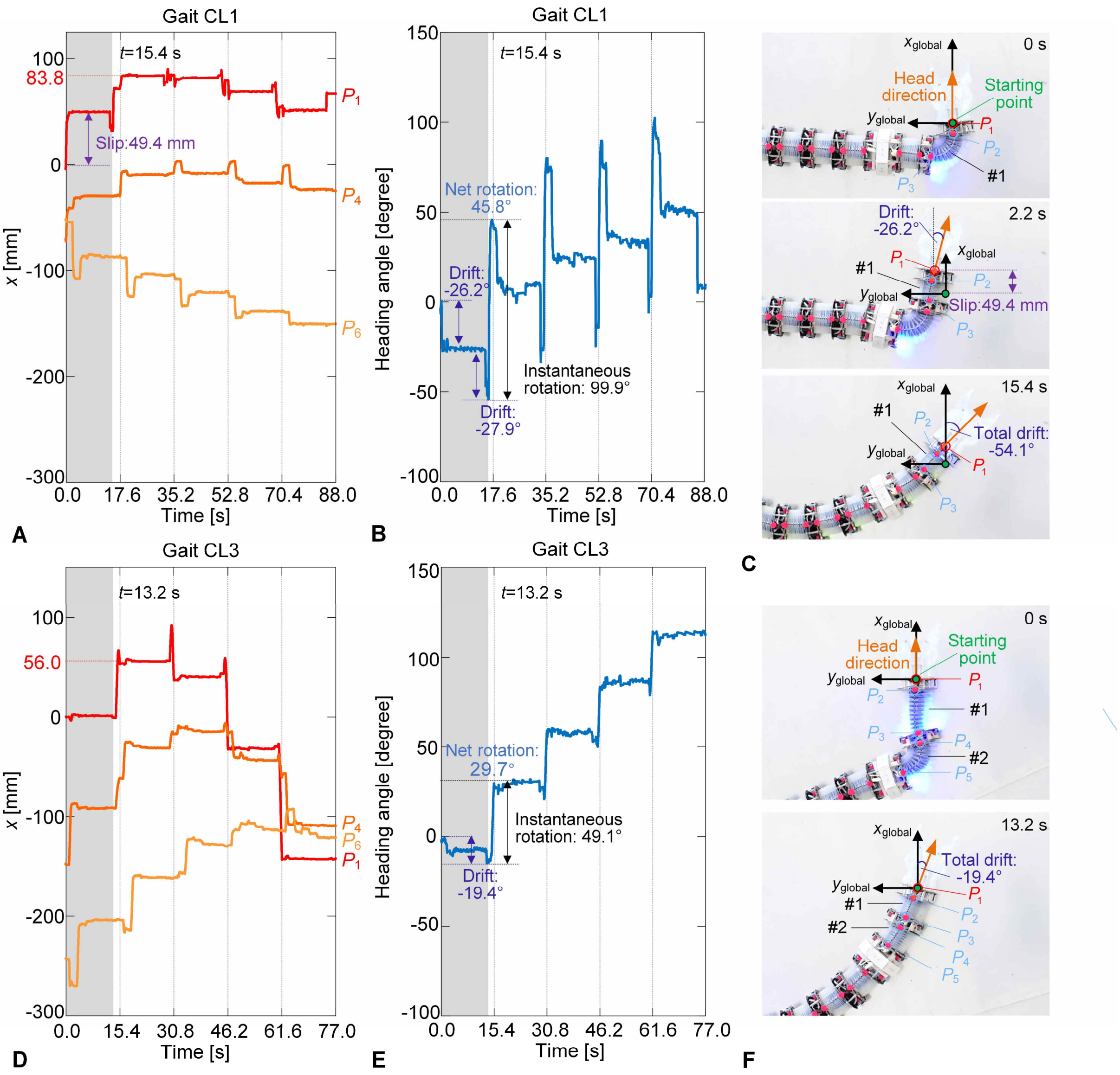

Figure S12. Experimental results of circular locomotion under gaits CL1 and CL3. A) and B) respectively show the time histories of the x-direction displacement of markers P_1, P_4, P_6, and heading angle under gait CL1. C) The screenshot of the robot's head slippage and rotation drift. D) and E) respectively show the time histories of the x-direction displacement of markers P_1, P_4, P_6, and heading angle under gait CL3. F) The screenshot of the robot's head rotation drift.

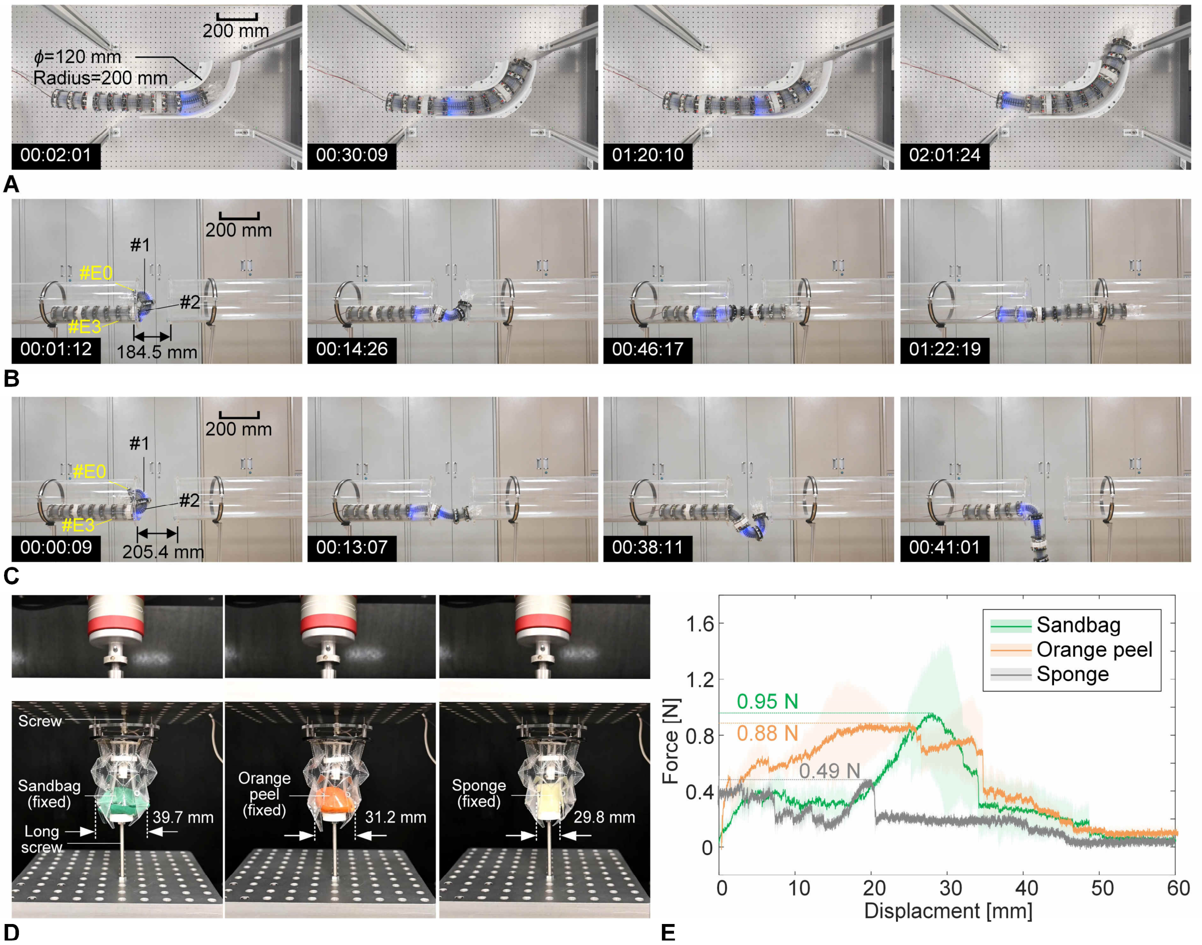

Figure S13. Quantitative evaluation of the robot's task performance. A) The snapshots of the robot traversing a curved pipe with a small bending radius. B) The snapshots from the test of traversable pipe discontinuities. C) The snapshots from the test of non-traversable pipe discontinuities. D) The experiment setup of the grasping force test. E) Grasping force–displacement curves of the gripping module for grasping the sponge, orange peel, and sandbag.

Supporting Tables

Table S1. Deformation states and corresponding valve activation commands

States	Driving mode
Axially-contracted	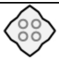
Axially-extended	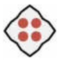
Upward-bent	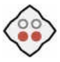
Downward-bent	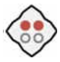
Rightward-bent	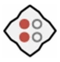
Leftward-bent	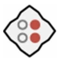
Upper-right-bent	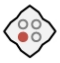
Lower-right-bent	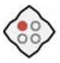
Upper-left-bent	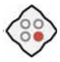
Lower-left-bent	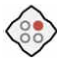

Table S2. Gait parameters and commands for earthworm-like rectilinear locomotion.

Gait #	Gait parameters $\{n_0,n_1,n_2,n_3,n_4,n_5,n_R\|n_p\}$	Crawling module commands (state vectors $\mathbf{s}^t$)	Electromagnet commands (electromagnet state vectors $\mathbf{e}^t$)
R1	{7,1,0,0,0,0,0|1}	Crawling module #<br>8 7 6 5 4 3 2 1<br>Time<br>t_0 0,0,0,0,0,0,0,1;<br>$t_0+\Delta t$ 0,0,0,0,0,0,1,0;<br>$t_0+2\Delta t$ 0,0,0,0,0,1,0,0;<br>$t_0+3\Delta t$ 0,0,0,0,1,0,0,0;<br>$t_0+4\Delta t$ 0,0,0,1,0,0,0,0;<br>$t_0+5\Delta t$ 0,0,1,0,0,0,0,0;<br>$t_0+6\Delta t$ 0,1,0,0,0,0,0,0;<br>$t_0+7\Delta t$ 1,0,0,0,0,0,0,0;<br>$t_0+8\Delta t$ 0,0,0,0,0,0,0,1;	Electromagnet module #<br>E8 E7 E6 E5 E4 E3 E2 E1 E0<br>t_0 0, 0, 0, 0, 0, 0, 0, 1, 0;<br>$t_0+\Delta t$ 0, 0, 0, 0, 0, 0, 0, 0, 1;<br>$t_0+2\Delta t$ 0, 0, 0, 0, 0, 0, 0, 0, 1;<br>$t_0+3\Delta t$ 0, 0, 0, 0, 0, 0, 0, 0, 1;<br>$t_0+4\Delta t$ 0, 0, 0, 0, 0, 0, 0, 0, 1;<br>$t_0+5\Delta t$ 0, 0, 0, 0, 0, 0, 0, 0, 1;<br>$t_0+6\Delta t$ 0, 0, 0, 0, 0, 0, 0, 0, 1;<br>$t_0+7\Delta t$ 0, 0, 0, 0, 0, 0, 0, 0, 1;<br>$t_0+8\Delta t$ 0, 0, 0, 0, 0, 0, 0, 1, 0;
R2	{7,1,0,0,0,0,0|2}	Crawling module #<br>8 7 6 5 4 3 2 1<br>Time<br>t_0 0,0,0,0,0,0,0,1;<br>$t_0+\Delta t$ 0,0,0,0,0,1,0,0;<br>$t_0+2\Delta t$ 0,0,0,1,0,0,0,0;<br>$t_0+3\Delta t$ 0,1,0,0,0,0,0,0;<br>$t_0+4\Delta t$ 0,0,0,0,0,0,0,1;	Electromagnet pair #<br>E8 E7 E6 E5 E4 E3 E2 E1 E0<br>Time<br>t_0 0, 0, 0, 0, 0, 0, 0, 1, 0;<br>$t_0+\Delta t$ 0, 0, 0, 0, 0, 0, 0, 0, 1;<br>$t_0+2\Delta t$ 0, 0, 0, 0, 0, 0, 0, 0, 1;<br>$t_0+3\Delta t$ 0, 0, 0, 0, 0, 0, 0, 0, 1;<br>$t_0+4\Delta t$ 0, 0, 0, 0, 0, 0, 0, 1, 0;
R3	{6,2,0,0,0,0,0|1}	Crawling module #<br>8 7 6 5 4 3 2 1<br>Time<br>t_0 0,0,0,0,0,0,1,1;<br>$t_0+\Delta t$ 0,0,0,0,0,1,1,0;<br>$t_0+2\Delta t$ 0,0,0,0,1,1,0,0;<br>$t_0+3\Delta t$ 0,0,0,1,1,0,0,0;<br>$t_0+4\Delta t$ 0,0,1,1,0,0,0,0;<br>$t_0+5\Delta t$ 0,1,1,0,0,0,0,0;<br>$t_0+6\Delta t$ 1,1,0,0,0,0,0,0;<br>$t_0+7\Delta t$ 0,0,0,0,0,0,1,1;	Electromagnet pair #<br>E8 E7 E6 E5 E4 E3 E2 E1 E0<br>Time<br>t_0 0, 0, 0, 0, 0, 0, 1, 0, 0;<br>$t_0+\Delta t$ 0, 0, 0, 0, 0, 0, 0, 0, 1;<br>$t_0+2\Delta t$ 0, 0, 0, 0, 0, 0, 0, 0, 1;<br>$t_0+3\Delta t$ 0, 0, 0, 0, 0, 0, 0, 0, 1;<br>$t_0+4\Delta t$ 0, 0, 0, 0, 0, 0, 0, 0, 1;<br>$t_0+5\Delta t$ 0, 0, 0, 0, 0, 0, 0, 0, 1;<br>$t_0+6\Delta t$ 0, 0, 0, 0, 0, 0, 0, 0, 1;<br>$t_0+7\Delta t$ 0, 0, 0, 0, 0, 0, 1, 0, 0;
R4	{6,2,0,0,0,0,0|2}	Crawling module #<br>8 7 6 5 4 3 2 1<br>Time<br>t_0 0,0,0,0,0,0,1,1;<br>$t_0+\Delta t$ 0,0,0,0,1,1,0,0;<br>$t_0+2\Delta t$ 0,0,1,1,0,0,0,0;<br>$t_0+3\Delta t$ 1,1,0,0,0,0,0,0;<br>$t_0+4\Delta t$ 0,0,0,0,0,0,1,1;	Electromagnet pair #<br>E8 E7 E6 E5 E4 E3 E2 E1 E0<br>Time<br>t_0 0, 0, 0, 0, 0, 0, 1, 0, 0;<br>$t_0+\Delta t$ 0, 0, 0, 0, 0, 0, 0, 0, 1;<br>$t_0+2\Delta t$ 0, 0, 0, 0, 0, 0, 0, 0, 1;<br>$t_0+3\Delta t$ 0, 0, 0, 0, 0, 0, 0, 0, 1;<br>$t_0+4\Delta t$ 0, 0, 0, 0, 0, 0, 1, 0, 0;

R5 {5,3,0,0,0,0,0|1}

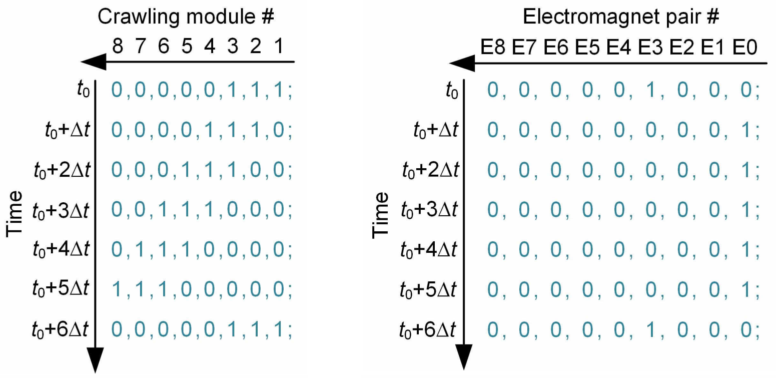

R6 {5,3,0,0,0,0,0|2}

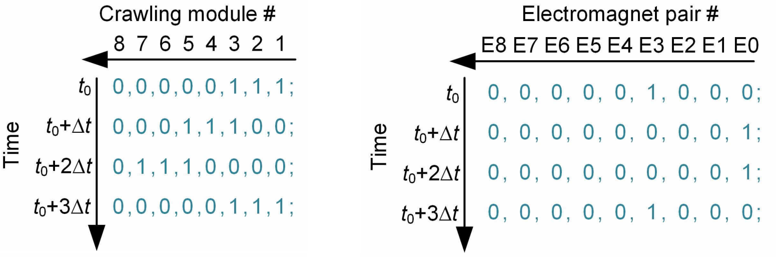

R7 {4,4,0,0,0,0,0|1}

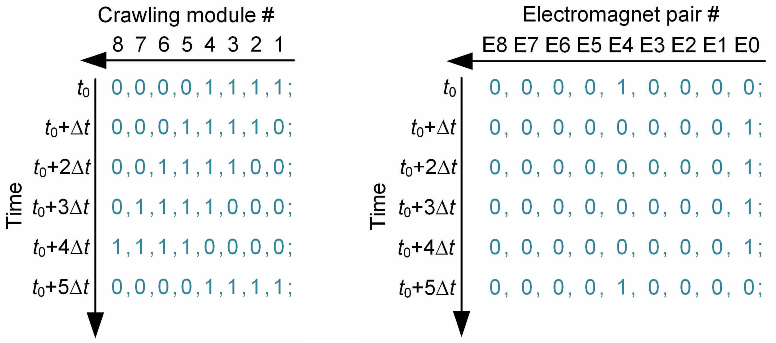

R8 {4,4,0,0,0,0,0|2}

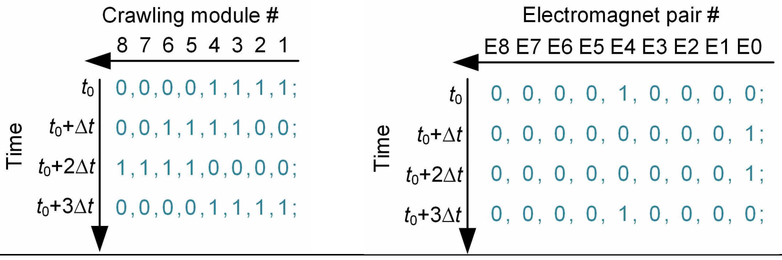

Table S3. Gait parameters and commands for earthworm-like sidewinding locomotion.

Gait #	Gait parameters $\{n_0,n_1,n_2,n_3,n_4,n_5,n_R \mid n_p\}$	Time	Crawling module commands (state vectors $\mathbf{s}^t$), Crawling module # 8 7 6 5 4 3 2 1	Electromagnet commands (electromagnet state vectors $\mathbf{e}^t$), Electromagnet pair # E8 E7 E6 E5 E4 E3 E2 E1 E0
S1	{6,0,0,0,1,1,0|1}	t_0	0,0,0,0,0,0,4,5;	0, 0, 0, 0, 0, 0, 0, 1, 0;
		$t_0+\Delta t$	0,0,0,0,0,4,5,0;	0, 0, 0, 0, 0, 0, 0, 0, 1;
		$t_0+2\Delta t$	0,0,0,0,4,5,0,0;	0, 0, 0, 0, 0, 0, 0, 0, 1;
		$t_0+3\Delta t$	0,0,0,4,5,0,0,0;	0, 0, 0, 0, 0, 0, 0, 0, 1;
		$t_0+4\Delta t$	0,0,4,5,0,0,0,0;	0, 0, 0, 0, 0, 0, 0, 0, 1;
		$t_0+5\Delta t$	0,4,5,0,0,0,0,0;	0, 0, 0, 0, 0, 0, 0, 0, 1;
		$t_0+6\Delta t$	4,5,0,0,0,0,0,0;	0, 0, 0, 0, 0, 0, 0, 0, 1;
		$t_0+7\Delta t$	0,0,0,0,0,0,4,5;	0, 0, 0, 0, 0, 0, 0, 0, 1;
S2	{6,0,0,0,1,1,0|2}	t_0	0,0,0,0,0,0,4,5;	0, 0, 0, 0, 0, 0, 1, 0, 0;
		$t_0+\Delta t$	0,0,0,0,4,5,0,0;	0, 0, 0, 0, 0, 0, 0, 0, 1;
		$t_0+2\Delta t$	0,0,4,5,0,0,0,0;	0, 0, 0, 0, 0, 0, 0, 0, 1;
		$t_0+3\Delta t$	4,5,0,0,0,0,0,0;	0, 0, 0, 0, 0, 0, 0, 0, 1;
		$t_0+4\Delta t$	0,0,0,0,0,0,4,5;	0, 0, 0, 0, 0, 0, 1, 0, 0;
S3	{5,1,0,0,1,1,0|1}	t_0	0,0,0,0,0,1,4,5;	0, 0, 0, 0, 0, 1, 0, 0, 0;
		$t_0+\Delta t$	0,0,0,0,1,4,5,0;	0, 0, 0, 0, 0, 0, 0, 0, 1;
		$t_0+2\Delta t$	0,0,0,1,4,5,0,0;	0, 0, 0, 0, 0, 0, 0, 0, 1;
		$t_0+3\Delta t$	0,0,1,4,5,0,0,0;	0, 0, 0, 0, 0, 0, 0, 0, 1;
		$t_0+4\Delta t$	0,1,4,5,0,0,0,0;	0, 0, 0, 0, 0, 0, 0, 0, 1;
		$t_0+5\Delta t$	1,4,5,0,0,0,0,0;	0, 0, 0, 0, 0, 0, 0, 0, 1;
		$t_0+6\Delta t$	4,5,0,0,0,0,0,0;	0, 0, 0, 0, 0, 1, 0, 0, 0;
S4	{5,1,0,0,1,1,0|2}	t_0	0,0,0,0,0,1,4,5;	0, 0, 0, 0, 0, 1, 0, 0, 0;
		$t_0+\Delta t$	0,0,0,1,4,5,0,0;	0, 0, 0, 0, 0, 0, 0, 0, 1;
		$t_0+2\Delta t$	0,1,4,5,0,0,0,0;	0, 0, 0, 0, 0, 0, 0, 0, 1;
		$t_0+3\Delta t$	0,0,0,0,0,1,4,5;	0, 0, 0, 0, 0, 1, 0, 0, 0;
S5	{4,2,0,0,1,1,0|1}	t_0	0,0,0,0,1,1,4,5;	0, 0, 0, 0, 0, 1, 0, 0, 0;
		$t_0+\Delta t$	0,0,0,1,1,4,5,0;	0, 0, 0, 0, 0, 0, 0, 0, 1;
		$t_0+2\Delta t$	0,0,1,1,4,5,0,0;	0, 0, 0, 0, 0, 0, 0, 0, 1;
		$t_0+3\Delta t$	0,1,1,4,5,0,0,0;	0, 0, 0, 0, 0, 0, 0, 0, 1;
		$t_0+4\Delta t$	1,1,4,5,0,0,0,0;	0, 0, 0, 0, 0, 0, 0, 0, 1;
		$t_0+5\Delta t$	0,0,0,0,1,1,4,5;	0, 0, 0, 0, 0, 1, 0, 0, 0;

S6	{4,2,0,0,1,1,0|2}	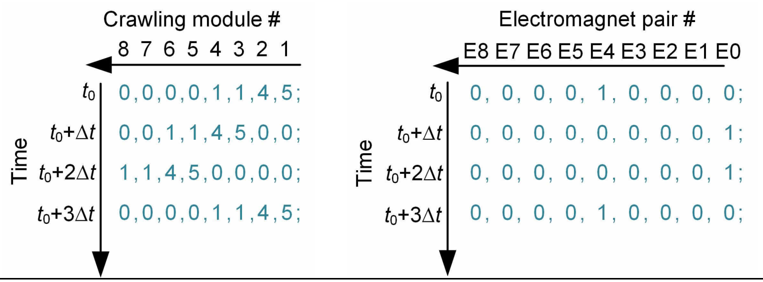

Crawling module #
8 7 6 5 4 3 2 1
Time
t0
t0+Δt
t0+2Δt
t0+3Δt
0,0,0,0,1,1,4,5;
0,0,1,1,4,5,0,0;
1,1,4,5,0,0,0,0;
0,0,0,0,1,1,4,5;
Electromagnet pair #
E8 E7 E6 E5 E4 E3 E2 E1 E0
0, 0, 0, 0, 1, 0, 0, 0, 0;
0, 0, 0, 0, 0, 0, 0, 0, 1;
0, 0, 0, 0, 0, 0, 0, 0, 1;
0, 0, 0, 0, 1, 0, 0, 0, 0;

Table S4. Gait parameters and commands for earthworm-like circular locomotion.

Gait #	Gait parameters $\{n_0,n_1,n_2,n_3,n_4,n_5,n_R \vert n_p\}$	Time	Crawling module commands (state vectors $\mathbf{s}^t$)<br>Crawling module # 8 7 6 5 4 3 2 1	Electromagnet commands (electromagnet state vectors $\mathbf{e}^t$)<br>Electromagnet pair # E8 E7 E6 E5 E4 E3 E2 E1 E0
CR1	{7,0,0,0,0,1,0|1}	t_0	0,0,0,0,0,0,0,4;	0, 0, 0, 0, 0, 0, 0, 1, 0;
		$t_0+\Delta t$	0,0,0,0,0,0,4,0;	0, 0, 0, 0, 0, 0, 0, 0, 1;
		$t_0+2\Delta t$	0,0,0,0,0,4,0,0;	0, 0, 0, 0, 0, 0, 0, 0, 1;
		$t_0+3\Delta t$	0,0,0,0,4,0,0,0;	0, 0, 0, 0, 0, 0, 0, 0, 1;
		$t_0+4\Delta t$	0,0,0,4,0,0,0,0;	0, 0, 0, 0, 0, 0, 0, 0, 1;
		$t_0+5\Delta t$	0,0,4,0,0,0,0,0;	0, 0, 0, 0, 0, 0, 0, 0, 1;
		$t_0+6\Delta t$	0,4,0,0,0,0,0,0;	0, 0, 0, 0, 0, 0, 0, 0, 1;
		$t_0+7\Delta t$	4,0,0,0,0,0,0,0;	0, 0, 0, 0, 0, 0, 0, 0, 1;
		$t_0+8\Delta t$	0,0,0,0,0,0,0,4;	0, 0, 0, 0, 0, 0, 0, 1, 0;
CR2	{7,0,0,0,0,1,0|2}	t_0	0,0,0,0,0,0,0,4;	0, 0, 0, 0, 0, 0, 0, 1, 0;
		$t_0+\Delta t$	0,0,0,0,0,4,0,0;	0, 0, 0, 0, 0, 0, 0, 0, 1;
		$t_0+2\Delta t$	0,0,0,4,0,0,0,0;	0, 0, 0, 0, 0, 0, 0, 0, 1;
		$t_0+3\Delta t$	0,4,0,0,0,0,0,0;	0, 0, 0, 0, 0, 0, 0, 0, 1;
		$t_0+4\Delta t$	0,0,0,0,0,0,0,4;	0, 0, 0, 0, 0, 0, 0, 1, 0;
CR3	{6,1,0,0,0,1,0|1}	t_0	0,0,0,0,0,0,4,1;	0, 0, 0, 0, 0, 0, 1, 0, 0;
		$t_0+\Delta t$	0,0,0,0,0,4,1,0;	0, 0, 0, 0, 0, 0, 0, 0, 1;
		$t_0+2\Delta t$	0,0,0,0,4,1,0,0;	0, 0, 0, 0, 0, 0, 0, 0, 1;
		$t_0+3\Delta t$	0,0,0,4,1,0,0,0;	0, 0, 0, 0, 0, 0, 0, 0, 1;
		$t_0+4\Delta t$	0,0,4,1,0,0,0,0;	0, 0, 0, 0, 0, 0, 0, 0, 1;
		$t_0+5\Delta t$	0,4,1,0,0,0,0,0;	0, 0, 0, 0, 0, 0, 0, 0, 1;
		$t_0+6\Delta t$	4,1,0,0,0,0,0,0;	0, 0, 0, 0, 0, 0, 0, 0, 1;
		$t_0+7\Delta t$	0,0,0,0,0,0,4,1;	0, 0, 0, 0, 0, 0, 1, 0, 0;
CR4	{6,1,0,0,0,1,0|2}	t_0	0,0,0,0,0,0,4,1;	0, 0, 0, 0, 0, 0, 1, 0, 0;
		$t_0+\Delta t$	0,0,0,0,4,1,0,0;	0, 0, 0, 0, 0, 0, 0, 0, 1;
		$t_0+2\Delta t$	0,0,4,1,0,0,0,0;	0, 0, 0, 0, 0, 0, 0, 0, 1;
		$t_0+3\Delta t$	4,1,0,0,0,0,0,0;	0, 0, 0, 0, 0, 0, 0, 0, 1;
		$t_0+4\Delta t$	0,0,0,0,0,0,4,1;	0, 0, 0, 0, 0, 0, 1, 0, 0;

		Crawling module # (8 7 6 5 4 3 2 1)	Electromagnet pair # (E8 E7 E6 E5 E4 E3 E2 E1 E0)
CL1	{7,0,1,0,0,0,0|1}	t_0: 0,0,0,0,0,0,0,5;<br>$t_0+\Delta t$: 0,0,0,0,0,0,5,0;<br>$t_0+2\Delta t$: 0,0,0,0,0,5,0,0;<br>$t_0+3\Delta t$: 0,0,0,0,5,0,0,0;<br>$t_0+4\Delta t$: 0,0,0,5,0,0,0,0;<br>$t_0+5\Delta t$: 0,0,5,0,0,0,0,0;<br>$t_0+6\Delta t$: 0,5,0,0,0,0,0,0;<br>$t_0+7\Delta t$: 5,0,0,0,0,0,0,0;<br>$t_0+8\Delta t$: 0,0,0,0,0,0,0,5;	t_0: 0, 0, 0, 0, 0, 0, 0, 1, 0;<br>$t_0+\Delta t$: 0, 0, 0, 0, 0, 0, 0, 0, 1;<br>$t_0+2\Delta t$: 0, 0, 0, 0, 0, 0, 0, 0, 1;<br>$t_0+3\Delta t$: 0, 0, 0, 0, 0, 0, 0, 0, 1;<br>$t_0+4\Delta t$: 0, 0, 0, 0, 0, 0, 0, 0, 1;<br>$t_0+5\Delta t$: 0, 0, 0, 0, 0, 0, 0, 0, 1;<br>$t_0+6\Delta t$: 0, 0, 0, 0, 0, 0, 0, 0, 1;<br>$t_0+7\Delta t$: 0, 0, 0, 0, 0, 0, 0, 0, 1;<br>$t_0+8\Delta t$: 0, 0, 0, 0, 0, 0, 0, 1, 0;
CL2	{7,0,1,0,0,0,0|2}	t_0: 0,0,0,0,0,0,0,5;<br>$t_0+\Delta t$: 0,0,0,0,0,5,0,0;<br>$t_0+2\Delta t$: 0,0,0,5,0,0,0,0;<br>$t_0+3\Delta t$: 0,5,0,0,0,0,0,0;<br>$t_0+4\Delta t$: 0,0,0,0,0,0,0,5;	t_0: 0, 0, 0, 0, 0, 0, 0, 1, 0;<br>$t_0+\Delta t$: 0, 0, 0, 0, 0, 0, 0, 0, 1;<br>$t_0+2\Delta t$: 0, 0, 0, 0, 0, 0, 0, 0, 1;<br>$t_0+3\Delta t$: 0, 0, 0, 0, 0, 0, 0, 0, 1;<br>$t_0+4\Delta t$: 0, 0, 0, 0, 0, 0, 0, 1, 0;
CL3	{6,1,1,0,0,0,0|1}	t_0: 0,0,0,0,0,0,5,1;<br>$t_0+\Delta t$: 0,0,0,0,0,5,1,0;<br>$t_0+2\Delta t$: 0,0,0,0,5,1,0,0;<br>$t_0+3\Delta t$: 0,0,0,5,1,0,0,0;<br>$t_0+4\Delta t$: 0,0,5,1,0,0,0,0;<br>$t_0+5\Delta t$: 0,5,1,0,0,0,0,0;<br>$t_0+6\Delta t$: 5,1,0,0,0,0,0,0;<br>$t_0+7\Delta t$: 0,0,0,0,0,0,5,1;	t_0: 0, 0, 0, 0, 0, 0, 1, 0, 0;<br>$t_0+\Delta t$: 0, 0, 0, 0, 0, 0, 0, 0, 1;<br>$t_0+2\Delta t$: 0, 0, 0, 0, 0, 0, 0, 0, 1;<br>$t_0+3\Delta t$: 0, 0, 0, 0, 0, 0, 0, 0, 1;<br>$t_0+4\Delta t$: 0, 0, 0, 0, 0, 0, 0, 0, 1;<br>$t_0+5\Delta t$: 0, 0, 0, 0, 0, 0, 0, 0, 1;<br>$t_0+6\Delta t$: 0, 0, 0, 0, 0, 0, 0, 0, 1;<br>$t_0+7\Delta t$: 0, 0, 0, 0, 0, 0, 1, 0, 0;
CL4	{6,1,1,0,0,0,0|2}	t_0: 0,0,0,0,0,0,5,1;<br>$t_0+\Delta t$: 0,0,0,0,5,1,0,0;<br>$t_0+2\Delta t$: 0,0,5,1,0,0,0,0;<br>$t_0+3\Delta t$: 5,1,0,0,0,0,0,0;<br>$t_0+4\Delta t$: 0,0,0,0,0,0,5,1;	t_0: 0, 0, 0, 0, 0, 0, 1, 0, 0;<br>$t_0+\Delta t$: 0, 0, 0, 0, 0, 0, 0, 0, 1;<br>$t_0+2\Delta t$: 0, 0, 0, 0, 0, 0, 0, 0, 1;<br>$t_0+3\Delta t$: 0, 0, 0, 0, 0, 0, 0, 0, 1;<br>$t_0+4\Delta t$: 0, 0, 0, 0, 0, 0, 1, 0, 0;

Table S5. Gait parameters and commands for inchworm-like two-anchor crawling and wheel rolling.

Gait #	Gait parameters $\{n_0,n_1,n_2,n_3,n_4,n_5,n_R \vert n_p\}$	Crawling module commands (state vectors $\mathbf{s}^t$)	Electromagnet commands (electromagnet state vectors $\mathbf{e}^t$)
I1	{0,4,2,2,0,0,0|0}	Crawling module #<br>8 7 6 5 4 3 2 1<br>Time<br>t_0 0,0,2,3,3,2,0,0;<br>$t_0+\Delta t$ 0,0,1,1,1,1,0,0;<br>$t_0+2\Delta t$ 0,0,2,3,3,2,0,0;	Electromagnet pair #<br>E8 E7 E6 E5 E4 E3 E2 E1 E0<br>Time<br>t_0 0, 0, 0, 0, 0, 0, 1, 0, 0;<br>$t_0+\Delta t$ 0, 0, 1, 0, 0, 0, 0, 0, 0;<br>$t_0+2\Delta t$ 0, 0, 0, 0, 0, 0, 1, 0, 0;
I2	{0,3,2,1,0,0,0|0}	Crawling module #<br>8 7 6 5 4 3 2 1<br>Time<br>t_0 0,0,0,2,3,2,0,0;<br>$t_0+\Delta t$ 0,0,0,1,1,1,0,0;<br>$t_0+2\Delta t$ 0,0,0,2,3,2,0,0;	Electromagnet pair #<br>E8 E7 E6 E5 E4 E3 E2 E1 E0<br>Time<br>t_0 0, 0, 0, 0, 0, 0, 1, 0, 0;<br>$t_0+\Delta t$ 0, 0, 1, 0, 0, 0, 0, 0, 0;<br>$t_0+2\Delta t$ 0, 0, 0, 0, 0, 0, 1, 0, 0;
RO1	{8,0,0,0,0,0,1|0}	/	/
RO2	{8,0,0,0,0,0,-1|0}	/	/

Table S6. Kinematic performance and environmental adaptability of different gaits

Locomotion mode	Gait #	Trajectory shape	Kinematic indices	Recommended application scenarios
Earthworm-like rectilinear locomotion	R1	Straight line	Velocity: 3.29 mm/s	Suitable for locomotion on open flat surfaces, in straight pipes with diameters no smaller than 120 mm, as well as in inclined and curved pipes.
	R2		Velocity: 5.95 mm/s	
	R3		Velocity: 6.41 mm/s	
	R4		Velocity: 11.28 mm/s	
	R5		Velocity: 9.55 mm/s	
	R6		Velocity: 18.76 mm/s	
	R7		Velocity: 8.68 mm/s	
	R8		Velocity: 15.37 mm/s	
Earthworm-like sidewinding locomotion	S1	Oblique line	Velocity: 8.71 mm/s<br>Incline angle: 86.31°	Suitable for oblique locomotion on open flat surfaces.
	S2		Velocity: 14.25 mm/s<br>Incline angle: 83.01°	
	S3		Velocity: 6.54 mm/s<br>Incline angle: 47.81°	
	S4		Velocity: 11.09 mm/s<br>Incline angle: 56.80°	
	S5		Velocity: 6.60 mm/s<br>Incline angle: 40.21°	
	S6		Velocity: 13.20 mm/s<br>Incline angle: 20.93°	
Earthworm-like circular locomotion	CR1	arc	Velocity: 2.38 mm/s<br>Radius: 92.21 mm	Suitable for curved locomotion or turning on open flat surfaces.
	CR2		Velocity: 4.61 mm/s<br>Radius: 118.04 mm	
	CR3		Velocity: 8.47 mm/s<br>Radius: 277.64 mm	
	CR4		Velocity: 13.74 mm/s<br>Radius: 262.75 mm	
	CL1		Velocity: 2.96 mm/s<br>Radius: 111.00 mm	
	CL2		Velocity: 4.17 mm/s<br>Radius: 101.24 mm	
	CL3		Velocity: 8.91 mm/s<br>Radius: 273.59 mm	
	CL4		Velocity: 14.97 mm/s<br>Radius: 256.44 mm	
Inchworm-like two-anchor crawling	I1	Straight line	Velocity: 33.29 mm/s	Suitable for rapid locomotion on open flat surfaces and in straight pipes with diameters no smaller than 200 mm.
	I2		Velocity: 5.95 mm/s	
Wheel rolling	RO1	Straight line	Velocity: 6.41 mm/s	Suitable for lateral locomotion on open flat surfaces.
	RO2		Velocity: 11.28 mm/s	

Supporting Movies

Movie S1. Deformation capability of the Yoshimura-origami structure

Movie S2. Locomotion tests for earthworm-like rectilinear peristaltic crawling

Movie S3. Locomotion tests for earthworm-like sidewinding peristaltic crawling

Movie S4. Locomotion tests for earthworm-like circular peristaltic crawling

Movie S5. Locomotion tests for inchworm-like two-anchor crawling and wheel-rolling

Movie S6. Robot field test in a complex industrial pipeline scenario

Movie S7. Quantitative evaluation of the robot's locomotion and task execution capabilities
